# Three-year follow-up analysis of phase 1/2 study on tirabrutinib in patients with relapsed or refractory primary central nervous system lymphoma

**DOI:** 10.1093/noajnl/vdae037

**Published:** 2024-04-22

**Authors:** Hajime Yonezawa, Yoshitaka Narita, Motoo Nagane, Kazuhiko Mishima, Yasuhito Terui, Yoshiki Arakawa, Katsunori Asai, Noriko Fukuhara, Kazuhiko Sugiyama, Naoki Shinojima, Arata Aoi, Ryo Nishikawa

**Affiliations:** Department of Neurosurgery, Kagoshima University Hospital, Kagoshima, Kagoshima, Japan; Department of Neurosurgery and Neuro-Oncology, National Cancer Center Hospital, Tokyo, Japan; Department of Neurosurgery, Kyorin University Faculty of Medicine, Mitaka, Tokyo, Japan; Department of Neuro-Oncology/Neurosurgery, Saitama Medical University International Medical Center, Hidaka, Saitama, Japan; Department of Hematology Oncology, The Cancer Institute Hospital, Japanese Foundation for Cancer Research, Tokyo, Japan; Department of Neurosurgery, Kyoto University Graduate School of Medicine, Kyoto, Kyoto, Japan; Department of Neurosurgery, Osaka International Cancer Institute, Osaka, Osaka, Japan; Department of Hematology, Tohoku University Hospital, Sendai, Miyagi, Japan; Department of Clinical Oncology & Neuro-oncology Program, Hiroshima University Hospital, Hiroshima, Hiroshima, Japan; Department of Neurosurgery, Kumamoto University Hospital, Kumamoto, Kumamoto, Japan; Department of Clinical Development, Ono Pharmaceutical Co., Ltd., Osaka, Osaka, Japan; Department of Neuro-Oncology/Neurosurgery, Saitama Medical University International Medical Center, Hidaka, Saitama, Japan

**Keywords:** BTKi, Bruton’s tyrosine kinase inhibitor, ONO-4059, PCNSL, primary central nervous system lymphoma, tirabrutinib

## Abstract

**Background:**

The ONO-4059-02 phase 1/2 study showed favorable efficacy and acceptable safety profile of tirabrutinib, a second-generation Bruton’s tyrosine kinase inhibitor, for relapsed/refractory primary central nervous system lymphoma (PCNSL). Here, we report the long-term efficacy and safety after a 3-year follow-up.

**Methods:**

Eligible patients were aged ≥ 20 years with histologically diagnosed PCNSL and KPS of ≥ 70. Patients received oral tirabrutinib once daily at 320 or 480 mg, or 480 mg under fasted conditions.

**Results:**

Between October 19, 2017, and June 13, 2019, 44 patients were enrolled: 33 and 9 had relapsed and refractory, respectively. The 320, 480, and 480 mg fasted groups included 20, 7, and 17 patients, respectively. The median follow-up was 37.1 months. The overall response rate was 63.6% (95% CI: 47.8–77.6) with complete response (CR), unconfirmed CR, and partial response in 9, 7, and 12 patients, respectively. The median duration of response (DOR) was 9.2 months, with a DOR rate of 19.8%; the median progression-free survival (PFS) and median overall survival (OS) were 2.9 months and not reached, respectively, with PFS and OS rates of 13.9% and 56.7%, respectively. Adverse events occurred in 38 patients (86.4%): grade ≥ 3 in 23 (52.3%) including 1 patient with grade 5 events. KPS and quality of life (QoL) scores were well maintained among patients receiving long-term treatment.

**Conclusions:**

The results demonstrated the long-term clinical benefit of tirabrutinib, with deep and durable response in a subset of patients and acceptable safety profile, while KPS and QoL scores were maintained.

**Key Points**

- The 3-year follow-up demonstrated long-term clinical benefit and safety of tirabrutinib for relapsed/refractory PCNSL.
- A fraction of patients achieved deep and durable responses to tirabrutinib.
- KPS and quality of life scores were generally maintained during the treatment.

**Importance of the Study**

Primary central nervous system lymphoma (PCNSL) is a type of non-Hodgkin lymphoma with poor prognosis, with the majority of patients relapsing under current therapeutic approaches. Current therapeutic approaches are insufficient to improve the clinical outcomes of PCNSL, especially those of relapsed/refractory cases. The ONO-4059-02 phase 1/2 study showed favorable efficacy and a manageable safety profile of tirabrutinib for relapsed/refractory PCNSL. Consequently, the use of tirabrutinib was approved in Japan, Korea, and Taiwan. However, the long-term efficacy and safety of tirabrutinib had remained unclear. Here, we updated the efficacy and safety of tirabrutinib with a 3-year follow-up. Overall, the results were consistent with the primary analysis, with a fraction of patients achieving durable and deep responses during this extended follow-up, demonstrating long-term clinical benefits of tirabrutinib. In addition, KPS and quality of life scores were maintained among patients who continued treatment. Further studies on a large scale and those involving a combination with existing treatment options, such as high-dose methotrexate-based therapy, are highly warranted.

Primary central nervous system lymphoma (PCNSL) is a rare type of non-Hodgkin lymphoma that primarily develops in brain, spinal cord, or eyes, and it accounts for 4% of intracranial tumors and 4%–6% of extranodal lymphomas.^1^ About 90% of PCNSL is diffuse large B-cell lymphoma (DLBCL), with most cases categorized into non-germinal center B-cell-like (non-GCB) type.^2,3^Among DLBCL, PCNSL shows a particularly poor prognosis.^4^

Currently, the standard of care for PCNSL has not been well established. Treatment with high-dose methotrexate (HD-MTX) followed by whole-brain radiation therapy (WBRT) had been formerly used as the main treatment option.^5^ As WBRT has been shown to cause delayed neurotoxicity,^6,7^ consolidation therapy with dose-reduced WBRT, autologous stem cell transplantation, or intensified chemotherapy have now replaced WBRT, especially for newly diagnosed PCNSL and/or relatively young patients.^8^ However, the prognosis of PCNSL remains poor, especially in patients with relapsed/refractory PCNSL.^9^ Therefore, new treatment options for those patients are highly warranted.

Non-GCB DLBCL often carries alterations in genes related to the B-cell receptor (BCR) signaling pathway, such as MYD88 and CD79B.^10^ Bruton’s tyrosine kinase (BTK) is a key mediator of the BCR signaling pathway and is considered as a potential therapeutic target of non-GCB DLBCL. Tirabrutinib is a second-generation BTK inhibitor with higher kinase specificity than that of the first-generation BTK inhibitor, ibrutinib.^11^ In the ONO-4059-02 phase 1/2 study, we evaluated safety, tolerability, efficacy, and pharmacokinetics of tirabrutinib in 44 Japanese patients with relapsed/refractory PCNSL who were administered oral tirabrutinib once daily (q.d.) at a dose of 320 or 480 mg, or of 480 mg under fasted condition. Overall, results of the study indicated safety and efficacy of tirabrutinib in relapsed/refractory PCNSL,^12^ leading to the approval of tirabrutinib for relapsed/refractory PCNSL in Japan (March 2020) and relapsed/refractory B-cell PCNSL in Korea (November 2021), and Taiwan (February 2022). A phase 2 study to evaluate efficacy and safety of tirabrutinib for PCNSL is now being conducted in the United States.^13^ Since efficacy and safety information is still limited for the long-term use of tirabrutinib in patients with relapsed/refractory PCNSL, we continuously followed patients in the ONO-4059-02 study. Here, we report the final analysis results of the ONO-4059-02 study with 3 years of follow-up.

## Materials and Methods

### Study Design

In the ONO-4059-02 open-label, single-arm, phase 1/2 study (Japanese Registry of Clinical Trials identifier, jRCT2080223590), patients with relapsed/refractory PCNSL were administered oral tirabrutinib q.d. at a dose of 320 or 480 mg, or 480 mg under fasted condition. The treatment was continued over 28-day cycles until disease progression or unacceptable toxicities were observed. On the basis of the results of the phase 1 dose-escalation part, the recommended dose was determined as 480 mg for the initiation of the phase 2 part. After the evaluation of dose-limiting toxicity in the phase 1 part, 1 patient who received 480 mg tirabrutinib died of adverse events (AEs; pneumocystis jirovecii pneumonia and interstitial lung disease), and 3 out of 4 patients who received 480 mg had grade 3 skin disorders; therefore, the dose for the phase 2 part was reduced to 320 mg. In another Japanese phase 1 study conducted concurrently with the current study, we found that in healthy Japanese adults, the maximum plasma concentration and area under the curve were increased after administration of tirabrutinib after a standard meal as compared to the administration under fasted conditions.^14^ Furthermore, 2 previous clinical studies showed that tirabrutinib was well tolerated in patients administered at the dose of 480 mg q.d. under fasted conditions.^15,16^ Thus, 17 patients were additionally recruited and received a dose of 480 mg under fasted conditions (Supplementary Figure S1).^12^ Other details of the study design were described previously.^12^ The study protocol was approved by the institutional review board of each site. This study was conducted under principles of the Declaration of Helsinki, the Good Clinical Practice guidelines, and local laws and regulations. All patients provided written informed consent.

### Patients

Eligible patients were those with age ≥ 20 years; histologically diagnosed, previously treated relapsed/refractory PCNSL; a brain lesion with the major axis > 1.0 cm; a KPS score ≥ 70; and expected lifetime ≥ 3 months. Patients without B-cell type PCNSL and those with systemic lesions were excluded. Other major exclusion criteria were detailed previously.^12^

### Assessments

The primary endpoint was the overall response rate (ORR) assessed by a central independent review committee, according to the International PCNSL Collaborative Group guidelines^17^ with categories of complete response (CR), unconfirmed CR (CRu), partial response (PR), stable disease (SD), and progressive disease (PD). The secondary endpoints included investigator-assessed ORR, best overall response (BOR), duration of response (DOR), time to response, progression-free survival (PFS), and overall survival (OS). PFS was defined as the time from the start of study treatment to PD or death, and OS was defined as the time from the start of study treatment to death. Tumor response was assessed using gadolinium-based brain MRI imaging at screening, day 28 of cycle 1, day 1 of cycles 3 through 25, and day 1 of every 4 cycles after cycle 25 during study treatment. Safety was assessed according to the National Cancer Institute Common Terminology Criteria for AEs version 4.0, Japanese translation version, by the Japan Clinical Oncology Group in patients receiving ongoing treatment with tirabrutinib. Allele-specific PCR-based genetic testing for mutations in MYD88, CD79A, CD79B, and CARD11 was performed in a central laboratory using patients’ DNA samples extracted from their tumor tissues that were obtained at the time of initial diagnosis.^12^ As exploratory endpoints, ORR and PFS per subgroup were assessed. Quality of life (QoL) of patients was assessed using the EuroQoL 5 dimensions 3-level (EQ-5D-3L) questionnaires and European Organisation for Research and Treatment of Cancer core QoL questionnaires (QLQ)-C30 and its brain tumor-specific module QLQ-BN20 at the same timings as for tumor response assessments. For patients with BOR of CR, CRu, PR, or SD who discontinued treatment for a safety reason, the QoL assessment was continued after treatment at regular intervals of 8 weeks where possible. Objective response, safety, and QoL were assessed in all patients enrolled.

### Statistical Analysis

The planned number of patients for enrollment was 3 or 6 for each cohort (320 or 480 mg) in the phase 1 part and 15 in the phase 2 part. Two-sided 95% confidence intervals (CIs) were calculated using the Clopper-Pearson method. The variables for the multivariate analysis of PFS were chosen with the step-wise method, and were evaluated with multivariate logistic regression. Safety was assessed in all patients who received ≥ 1 dose of study treatment. Within the safety set, those who had ≥ 1 measurable lesion before the start of treatment and had ≥ 1 centrally assessed BOR were assessed for the efficacy endpoints. All statistical analysis was performed using SAS^®^ version 9.4 (SAS Institute Inc., Cary, NC). For QoL assessment, mean and standard deviation for the changes from baseline in the QLQ-C30 functioning scales, QLQ-C30 or QLQ-BN20 symptoms scales, EQ-5D visual analog scales, and EQ-5D index score were calculated. The MID criteria employed in the current analysis were described previously.^18^ Differences in each score from their baseline were assessed by means of Dunnett’s test. The significance level was set at *P* = .05.

## Results

### Patient Characteristics and Duration of Treatment

Between October 19, 2017, and June 13, 2019, we enrolled 44 patients, including 33 and 9 patients with relapsed and refractory, respectively. The 320, 480, and 480 mg fasted groups included 20, 7, and 17 patients, respectively. The median follow-up period was 37.1 (range, 1.4–52.2) months in the overall population, 37.9 (range, 4.8–52.2) months in the 320 mg group, 48.6 (range, 1.4–50.2) months in the 480 mg group, and 36.8 (range, 2.9–40.1) months in the 480 mg fasted group (Table 1). Patient characteristics at baseline are summarized in Table 1. In the overall population, the median age was 60.0 years (range, 29–86) and the median for KPS scores was 80.0 (range, 70–100). The median number of previous lines of treatment was 2.0 (range, 1–14). All patients had previously received MTX and 29 patients (65.9%) had received WBRT (Table 1). The median duration of treatment was 2.7 (range, 0.8–46.9) months in the overall population, 2.3 (range, 0.9–46.9) months in the 320 mg group, 11.1 (range, 0.8–29.6) months in the 480 mg group, and 7.4 (range, 0.9–39.6) months in the 480 mg fasted group (Supplementary Table S1). The number of patients who continued treatment with tirabrutinib for 6 months, 1, 2, or 3 years was 20, 15, 8, and 6, respectively. Five patients (11.4%) had received treatment until discontinuation at study completion (Supplementary Table S1). Of those, 2 patients started to receive marketed tirabrutinib shortly thereafter. Major reasons for treatment discontinuation were disease progression (31 patients; 70.5%) and AEs (4 patients; 9.1%; Supplementary Table S1).

**Table 1.** Patient Characteristics at Baseline and Follow-up Periods

	All *n* = 44	320 mg *n* = 20	480 mg *n* = 7	480 mg fasted *n* = 17
Male, *n* (%)	24 (54.5)	14 (70.0)	4 (57.1)	6 (35.3)
Age, years, median (range)	60.0 (29–86)	59.5 (41–86)	54.0 (42–75)	65.0 (29–85)
KPS scores, median (range)	80.0 (70–100)	85.0 (70–100)	90.0 (70–100)	70.0 (70–100)
Number of previous lines of				
treatment, *n* (%)				
1	18 (40.9)	9 (45.0)	2 (28.6)	7 (41.2)
2–3	16 (36.4)	5 (25.0)	3 (42.9)	8 (47.1)
≥4	10 (22.7)	6 (30.0)	2 (28.6)	2 (11.8)
Median (range)	2.0 (1–14)	2.0 (1–6)	2.0 (1–14)	2.0 (1–5)
Previous treatment, *n* (%)				
Methotrexate	44 (100.0)	20 (100.0)	7 (100.0)	17 (100.0)
Cytarabine	21 (47.7)	7 (35.0)	4 (57.1)	10 (58.8)
Rituximab	26 (59.1)	13 (65.0)	3 (42.9)	10 (58.8)
WBRT	29 (65.9)	14 (70.0)	5 (71.4)	10 (58.8)
Surgery	16 (36.4)	8 (40.0)	2 (28.6)	6 (35.3)
ASCT	7 (15.9)	2 (10.0)	1 (14.3)	4 (23.5)
Disease status, *n* (%)				
Relapse	33 (75.0)	17 (85.0)	2 (28.6)	14 (82.4)
Refractory	9 (20.5)	3 (15.0)	3 (42.9)	3 (17.6)
Unknown	2 (4.5)	0	2 (28.6)	0
Relapse (MTX)	33 (75.0)	16 (80.0)	3 (42.9)	14 (82.4)
Refractory (MTX)	10 (22.7)	4 (20.0)	3 (42.9)	3 (17.6)
Relapse (rituximab)	20 (45.5)	10 (50.0)	2 (28.6)	8 (47.1)
Refractory (rituximab)	5 (11.4)	2 (10.0)	1 (14.3)	2 (11.8)
Oncogenic mutation, *n* (%)				
CARD11	17 (38.6)	3 (15.0)	2 (28.6)	12 (70.6)
CD79B	18 (40.9)	10 (50.0)	0	8 (47.1)
MYD88	32 (72.7)	15 (75.0)	6 (85.7)	11 (64.7)
Median follow-up period, months, (range)	37.1 (1.4–52.2)	37.9 (4.8–52.2)	48.6 (1.4–50.2)	36.8 (2.9–40.1)

WBRT, whole-brain radiotherapy; ASCT, autologous stem cell transplantation; MTX, methotrexate.

### Efficacy

**Figure 1.**
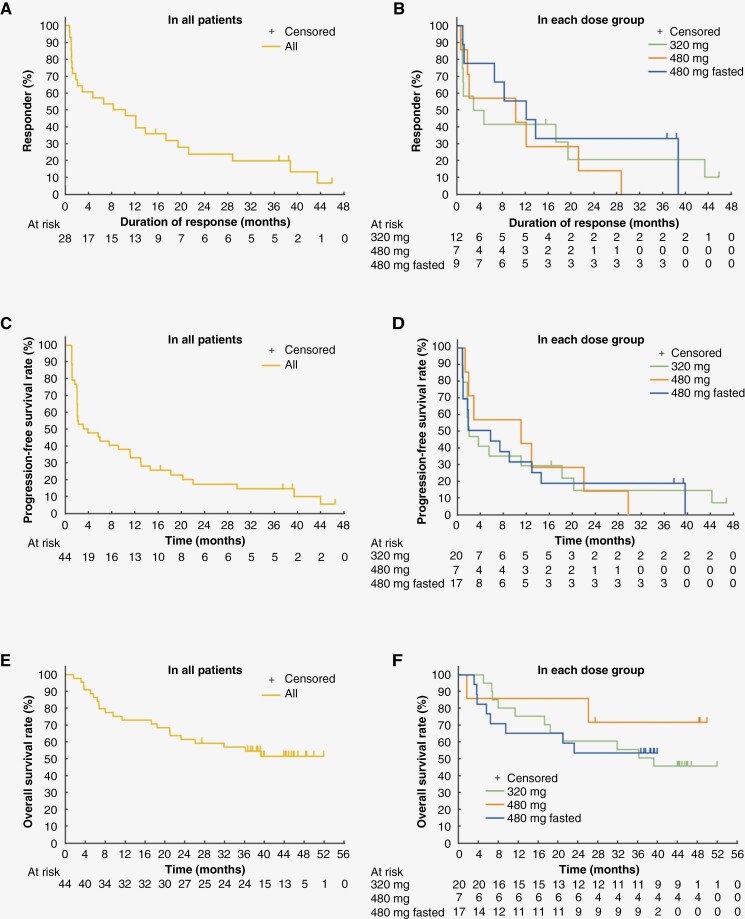
Efficacy outcomes of tirabrutinib treatment. The Kaplan–Meier curves of (A, B) duration of response (DOR), (C, D) progression-free survival (PFS), and (E, F) overall survival (OS) in all patients and each dose group are shown.

In the overall population, 28 patients achieved response to treatment with tirabrutinib (ORR, 63.6%; 95% CI: 47.8–77.6): 12 of the 20 patients in the 320 mg group (ORR, 60.0%; 95% CI: 36.1–80.9), 7 of the 7 patients in the 480 mg group (ORR, 100%; 95% CI: 59.0–100), and 9 of the 17 patients in the 480 mg fasted group (ORR, 52.9%; 95% CI: 27.8–77.0). Of note, after the previous 9.1 months follow-up,^12^ 1 patient had an improvement of BOR, with a shift from PR to CR, and 5 patients with CRu were confirmed to have CR. The median time to response was 0.9 months in both the overall population and each dose group (Supplementary Table S2). The median DOR was 9.2 (95% CI: 1.7–17.2) months in the overall population, 3.7 (95% CI: 0.9–19.4) months in the 320 mg group, 10.2 (95% CI: 0.6–21.2) months in the 480 mg group, and 12.1 (95% CI: 0.9–not reached) months in the 480 mg fasted group (Figure 1 and Supplementary Table S2). The 3-year DOR rate was 19.8% in the overall population, 20.8% in the 320 mg group, 0% in the 480 mg group, and 33.3% in the 480 mg fasted group. The median PFS was 2.9 (95% CI: 1.8–11.1) months in the overall population, 2.1 (95% CI: 1.8–18.2) months in the 320 mg group, 11.1 (95% CI: 1.4–22.0) months in the 480 mg group, and 5.8 (95% CI: 1.0–13.0) months in the 480 mg fasted group. The 3-year PFS rate was 13.9% in the overall population, 14.7% in the 320 mg group, 0% in the 480 mg group, and 19.0% in the 480 mg fasted group (Figure 1 and Supplementary Table S3). The median OS was not reached (95% CI: 21.0–not reached) in the overall population, 37.9 (95% CI: 11.2–not reached) months in the 320 mg group, not reached (95% CI: 1.4 months–not reached) in the 480 mg group, and not reached (95% CI: 5.5 months–not reached) in the 480 mg fasted group. The 3-year OS rate was 56.7% in the overall population, 55.0% in the 320 mg group, 71.4% in the 480 mg group, and 52.9% in the 480 mg fasted group (Figure 1 and Supplementary Table S3). Supplementary Figure S2 summarizes treatment course and survival outcomes of each patient. A total of 24 patients survived for 3 years from the start of tirabrutinib treatment, including 6 patients with ≥ 3 years of administration. Regarding survival outcomes by tumor response, 17 of the 28 patients (60.7%) who had a response to tirabrutinib (BOR of CR [*n* = 9], CRu [*n* = 3], or PR [*n* = 5]) and 7 of the 16 patients (43.8%) with BOR of SD (*n* = 3) or PD (*n* = 4) survived for 3 years.

### Subsequent Therapy and Survival Outcomes

A total of 42 patients (95.5%) terminated treatment (including those who completed study treatment) and 2 patients received ongoing treatment with marketed tirabrutinib. Of 42 patients who terminated tirabrutinib treatment, 32 (72.7%) started to receive subsequent treatment, including 28 (63.6%) who had disease progression under the treatment with tirabrutinib. The most frequent subsequent treatment was systemic therapy (25 patients [56.8%]), which included HD-MTX-based therapy in 18 patients (40.9%), and WBRT in 10 patients (22.7%; Table 2 and Supplementary Figure S2). Of note, 3-year survivors who had BOR of SD or PD (5 of the 7) had received subsequent treatment (Supplementary Figure S2). Patients who received HD-MTX-based therapy and those who received WBRT had 3-year OS rates of 61.1% and 40.0%, respectively (Supplementary Figure S3 and Table S4).

**Table 2.** Summary of Subsequent Therapy

	All	320 mg	480 mg	480 mg fasted
	*n* = 44	*n* = 20	*n* = 7	*n* = 17
Any subsequent therapy	32 (72.7)	14 (70.0)	6 (85.7)	12 (70.6)
WBRT	10 (22.7)	4 (20.0)	1 (14.3)	5 (29.4)
Surgery	1 (2.3)	0	0	1 (5.9)
HCT-ASCT	2 (4.5)	2 (10.0)	0	0
Systemic therapy	25 (56.8)	13 (65.0)	6 (85.7)	6 (35.3)
Rituximab	19 (43.2)	11 (55.0)	4 (57.1)	4 (23.5)
Methotrexate	18 (40.9)	9 (45.0)	4 (57.1)	5 (29.4)
Vincristine	14 (31.8)	6 (30.0)	4 (57.1)	4 (23.5)
Procarbazine	12 (27.3)	5 (25.0)	4 (57.1)	3 (17.6)
Cytarabine	7 (15.9)	5 (25.0)	0	2 (11.8)
Tirabrutinib rechallenge	3 (6.8)	1 (5.0)	1 (14.3)	1 (5.9)
Etoposide	3 (6.8)	2 (10.0)	1 (14.3)	0
Cisplatin	2 (4.5)	2 (10.0)	0	0
Methylprednisolone	1 (2.3)	1 (5.0)	0	0
Ifosfamide	1 (2.3)	1 (5.0)	0	0
Bevacizumab	1 (2.3)	1 (5.0)	0	0
CAR-T cellular therapy	1 (2.3)	0	1 (14.3)	0
Doxorubicin	1 (2.3)	0	0	1 (5.9)
Busulfan	1 (2.3)	1 (5.0)	0	0
Cyclophosphamide	1 (2.3)	0	0	1 (5.9)
Melphalan	1 (2.3)	1 (5.0)	0	0
Prednisolone	1 (2.3)	1 (5.0)	0	0
Ranimustine	1 (2.3)	1 (5.0)	0	0
Thiotepa	1 (2.3)	1 (5.0)	0	0
Tirabrutinib, ongoing	2 (4.5)	1 (5.0)	0	1 (5.9)
None	10 (22.7)	5 (25.0)	1 (14.3)	4 (23.5)

*n* (%) is shown. CAR-T, chimeric antigen receptor-T cell; HCT-ASCT, high-dose chemotherapy followed by autologous stem cell transplantation; WBRT, whole-brain radiotherapy.

### Efficacy by Subgroups

Subgroup analysis of ORR by patient characteristics showed generally comparable efficacy of tirabrutinib across patient backgrounds, except for several subgroups in which the ORR tended to be lower than that in the overall population: Those who were refractory after the last treatment (ORR, 33.3%), those who underwent ≥ 4 previous lines of chemotherapies (ORR, 33.3%), and those who had a history of high-dose chemotherapy followed by autologous stem cell transplantation (HCT-ASCT) for PCNSL (ORR, 28.6%), high tumor burden (ORR, 42.9%), or gene mutations of both CD79B and MYD88 (ORR, 40.0%; Supplementary Figure S4). No marked difference was observed in ORR, DOR, and PFS between patients with and without intraocular lymphoma and between those with and without CSF positivity (data not shown). No apparent difference was found in PFS and DOR between patients with either CD79B or MYD88 mutation and those with CD79B and MYD88 double mutation (data not shown). Subgroup analysis of PFS at 12 months suggested KPS as an independent factor that might have affected the PFS rates, with an odds ratio of PFS events (patients with KPS of 70–80 vs. 90–100) in the univariate and multivariate analysis being 5.999 (95% CI: 1.333–26.996; *P*-value, .0196) and 5.993 (95% CI: 1.202–29.876; *P*-value, .0289), respectively (Supplementary Table S5). We divided patients into 3 groups: Those with PFS of 0–3, 3–24, and > 24 months. Patients with PFS of 0–3 months, as compared to the other 2 groups, had lower KPS scores and higher tumor burden and included those who were refractory to last rituximab therapy for PCNSL. The ORR for patients with PFS of 0–3 months was 41.7%, with none of them achieving CR/CRu. The medians for OS were 14.2 months (95% CI: 6.2 months–not reached), not reached (95% CI: 23.3 months–not reached), and not reached (95% CI: not reached–not reached), in patients with PFS of 0–3, 3–24, and > 24 months, respectively. Within the patients with PFS of 0–3 months, the median interval between diagnosis of PCNSL and enrollment in this study was shorter in those with OS of < 12 months than in those with OS of ≥ 12 months (16.8 vs. 40.8 months).

### Safety

The incidence of AEs is summarized in Table 3. Any-grade AEs occurred in 38 patients (86.4%) and grade ≥ 3 AEs occurred in 23 patients (52.3%). The most frequent any-grade AEs were rash (36.4%), neutropenia (27.3%), leukopenia (25.0%), and lymphopenia (18.2%). The most frequent grade ≥ 3 AEs were neutropenia (9.1%), leukopenia (9.1%), lymphopenia (6.8%), and erythema multiforme (6.8%). One patient (2.3%) had fatal (grade 5) AEs, pneumocystis jirovecii pneumonia and interstitial lung disease, 32 days after starting 480 mg tirabrutinib. No patients had atrial fibrillation of any grade. Regarding treatment-related AEs (TRAEs) of special interest for tirabrutinib, such as skin-related disorders, cytopenia, infection, and diarrhea, most patients experienced any of such events within 6 months from the initiation of tirabrutinib treatment (Supplementary Figure S5). Most of the TRAEs of special interest resolved or were resolving, and most patients with skin-related disorders and infection received medical interventions for the events (Supplementary Table S6). TRAEs of special interest that first occurred beyond 6 months were grade 2 infection in 2 patients (at 6.1 months and 11.4 months from the start of treatment with tirabrutinib, respectively), who had treatment interruption and received anti-infective drug; and grade 2 cytopenia in 1 patient (at 26.7 months from the start of treatment with tirabrutinib) who continued treatment: All resolved during the study.

**Table 3.** Summary of Adverse Events

	All *n* = 44		320 mg *n* = 20		480 mg *n* = 7		480 mg fasted *n* = 17	
	Any grade	Grade ≥ 3	Any grade	Grade ≥ 3	Any grade	Grade ≥ 3	Any grade	Grade ≥ 3
Any adverse events	38 (86.4)	23 (52.3)	16 (80.0)	7 (35.0)	7 (100)	5 (71.4)*	15 (88.2)	11 (64.7)
Skin								
Rash	16 (36.4)	1 (2.3)	7 (35.0)	0	2 (28.6)	0	7 (41.2)	1 (5.9)
Erythema multiforme	5 (11.4)	3 (6.8)	2 (10.0)	1 (5.0)	3 (42.9)	2 (28.6)	0	0
Drug eruption	4 (9.1)	2 (4.5)	2 (10.0)	1 (5.0)	1 (14.3)	1 (14.3)	1 (5.9)	0
Hematologic								
Neutropenia	12 (27.3)	4 (9.1)	5 (25.0)	2 (10.0)	2 (28.6)	0	5 (29.4)	2 (11.8)
Leukopenia	11 (25.0)	4 (9.1)	3 (15.0)	2 (10.0)	3 (42.9)	0	5 (29.4)	2 (11.8)
Lymphopenia	8 (18.2)	3 (6.8)	3 (15.0)	0	3 (42.9)	2 (28.6)	2 (11.8)	1 (5.9)
Thrombocytopenia	5 (11.4)	0	1 (5.0)	0	3 (42.9)	0	1 (5.9)	0
Anemia	4 (9.1)	0	1 (5.0)	0	1 (14.3)	0	2 (11.8)	0
Increased blood bilirubin	4 (9.1)	1 (2.3)	4 (20.0)	1 (5.0)	0	0	0	0
Gastrointestinal								
Constipation	6 (13.6)	0	1 (5.0)	0	1 (14.3)	0	4 (23.5)	0
Stomatitis	5 (11.4)	0	3 (15.0)	0	1 (14.3)	0	1 (5.9)	0
Nausea	5 (11.4)	1 (2.3)	2 (10.0)	0	1 (14.3)	0	2 (11.8)	1 (5.9)
Vomiting	4 (9.1)	0	1 (5.0)	0	0	0	3 (17.6)	0
Infection								
Epipharyngitis	5 (11.4)	0	3 (15.0)	0	0	0	2 (11.8)	0
Others								
Headache	5 (11.4)	0	2 (10.0)	0	2 (28.6)	0	1 (5.9)	0

*n* (%) is shown. *Included 1 patient who had grade 5 adverse events (pneumocystis jirovecii pneumonia and interstitial lung disease) 32 days after starting tirabrutinib 480 mg.

### Karnofsky Performance Status and Quality of Life

We assessed temporal change in KPS scores and QoL scores in the overall population and in each dosage group (Supplementary Figure S6–9). The KPS score, as well as the score of QoL questionnaire items that are recognized as of importance for patients with PCNSL (global health status/QoL, emotional functioning, physical functioning, cognitive functioning, social functioning, constipation, and fatigue), were generally maintained in patients who continuously received treatment with tirabrutinib. In 22 of the 32 patients (68.8%) who had received subsequent treatment after tirabrutinib, favorable KPS scores (≥70) were maintained at discontinuation of tirabrutinib (data not shown).

## Discussion

**Figure 2.**
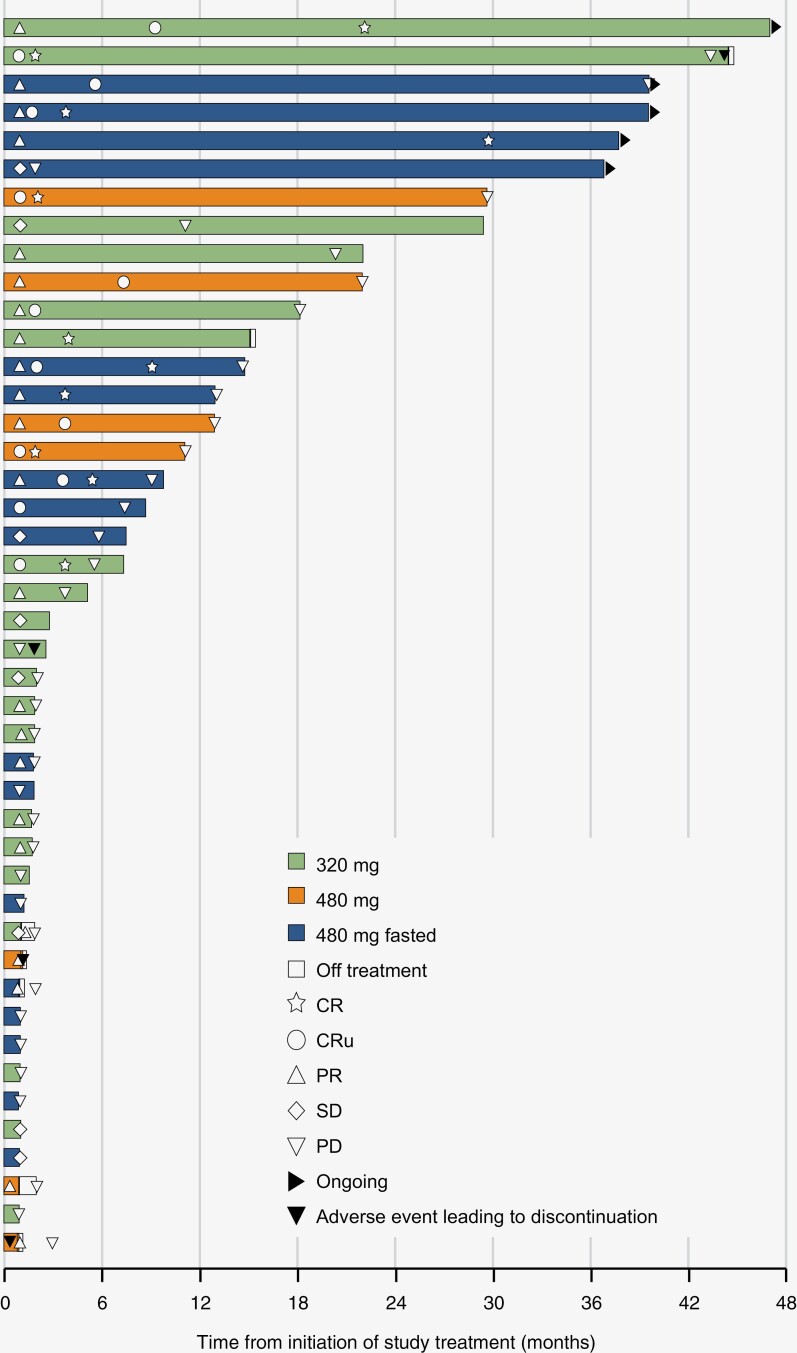
Treatment course and outcomes in each patient. The swimmer plot shows tumor response, duration of treatment, treatment status, and occurrence of adverse events that led to discontinuation of study drugs in all patients. BOR is per central assessment and duration of treatment is per investigators’ assessment. ^*^The patient developed an adverse event on a date on which he or she had centrally confirmed PD. CR, complete response; CRu, unconfirmed complete response; PR, partial response; q.d., once daily.

In this 3-year follow-up, we updated the efficacy and safety results of the phase 1/2 study of tirabrutinib in patients with relapsed/refractory PCNSL. Between the previous 9.1 months follow-up (CR in 4 patients, CRu in 11, PR in 13; ORR, 63.6%; CR/CRu, 34.1%) and the current 37.1 months follow-up (CR in 9 patients, CRu in 7, PR in 12; ORR, 63.6%; CR/CRu, 36.4%), 1 patient had improved BOR (PR to CR), while 5 patients with CRu being confirmed to have CR. As evidenced by the fact that treatment was discontinued mostly due to disease progression, not AEs, the manageable safety profile of tirabrutinib allowed patients to receive treatment for a long period, which may have contributed to the PR to CR transition. The facts that 80% (12 of the 15) of patients with CR/CRu continued treatment for ≥ 12 months (Figure 2) and that no patients with PFS of < 3 months had CR/CRu suggest that achieving CR/CRu is associated with longer DOR and PFS. This could have largely contributed to the discrepancy between DOR and PFS in the overall population (9.2 and 2.9 months, respectively), since the proportion of patients with CR/CRu included differs in each analysis. Further, to the best of our knowledge, this is one of few reports following treatment outcomes of BTK inhibitor monotherapy in patients with relapsed/refractory PCNSL for more than 3 years, which can be informative for clinical practice.

Monotherapy with other drugs (HD-MTX, temozolomide, topotecan, high-dose cytarabine, rituximab, temsirolimus, pemetrexed, and bendamustine) showed ORRs of 30.6%–90.9% (median, 51.0%) and CR/CRu rate of 0%–72.7% (median, 26.2%) in relapsed/refractory PCNSL.^9,19^ Ibrutinib, a first-generation BTK inhibitor, showed an ORR of 52% (CR/CRu, 19%).^20^ The current ORR (63.6%) and CR/CRu rate (36.4%) with tirabrutinib were comparable to those results. In the current study, the median DOR was 9.2 months and 12.1 months in the overall population and the 480 mg fasted group (approved dose), respectively; the median PFS was 2.9 and 5.8 months in the overall population and the 480 mg fasted group, respectively; the median OS was not reached in both overall population and the 480 mg fasted group; and the 36-month OS rate was 56.7% and 52.9% in the overall population and the 480 mg fasted group, respectively. The other monotherapy regimens showed comparable efficacy, in which medians for PFS were 1.9–25.8 months and medians for OS were 3.7–61.9 months^9,19^; ibrutinib showed a median DOR of 6.3 months, a median PFS of 4.8 months, and a median OS of 19.2 months.^20^ Taken together, tirabrutinib demonstrated clinical benefits in ORR, PFS, and OS that were comparable to monotherapy options for relapsed/refractory PCNSL. The favorable efficacy of tirabrutinib might be attributable to brain disposition of tirabrutinib. When patients were administered oral tirabrutinib at 320 or 480 mg q.d., trough plasma concentrations of tirabrutinib on day 28 were 16.3 ± 7.71 ng/mL and 77.0 ± 28.5 ng/mL, respectively,^12^ which might have attributed to the longer DOR in the 480 mg group than the 320 groups. Given that the protein binding ratio of tirabrutinib in serum is 92%,^14^ the unbound brain-to-plasma concentration ratios (K_p,uu,brain_) of tirabrutinib were 1.68 and 2.27 for the doses of 320 and 480 mg q.d., respectively. Based on these ratios, tirabrutinib is suggested to be well distributed in the brain through the blood-brain barrier.

Of the patients included in this study, 40.9% received subsequent HD-MTX-based therapy as subsequent treatment after discontinuation of tirabrutinib, which might have been allowed with the favorable KPS score (≥70) maintained in the majority of patients (68.8%) at discontinuation of tirabrutinib. Subsequent treatment might also have contributed to the survival outcomes of patients who had unfavorable BOR but survived for 3 years: Most 3-year survivors who had BOR of SD or PD had received subsequent treatment. PCNSL has a high chemosensitivity,^21^ but little is known about the treatment course and outcomes after disease progression in relapsed/refractory PCNSL. The outcomes of patients who progressed after tirabrutinib treatment and who were switched to a subsequent regimen would be useful information when strategizing a treatment plan for PCNSL. In general, acquisition of resistance to anti-cancer agents (including that of molecularly targeted drugs) involves mutations in components of signaling pathways, which often reduce effectiveness of subsequent regimens via cross-resistance.^22^ It is thus worth noting that subsequent HD-MTX-based therapy possibly contributed to the OS results of the current study. In addition, in PCNSL, it has been observed that tumors acquire new genetic mutations or phenotypes upon treatment^23,24^; thus, it is also possible that switching to a drug with a different mechanism of action contributes to widening treatment options for relapsed cases, including re-challenges.

Regarding safety, the most frequent AEs were rash, neutropenia, leukopenia, and lymphopenia; those were mostly grade 1 or 2, and the incidence was most frequent within 1 month from the initiation of tirabrutinib. Although TRAEs of special interest mostly occurred in the early phase after starting administration, 3 patients experienced TRAEs (infection or cytopenia) after 6 months of administration; therefore, careful monitoring may be required for the long-term use of BTK inhibitors.

KPS and QoL scores in the current results were comparable to those reported previously with the median follow-up period of 14.9 months,^18^ and were maintained during the long-term treatment, suggesting that the use of tirabrutinib did not negatively affect those scores during continuous administration. The KPS score has been identified as a prognostic factor in PCNSL patients.^25,26^ Similarly, in subgroup analysis of PFS in the current study, we identified the KPS score as a factor associated with favorable efficacy of tirabrutinib, suggesting that tirabrutinib may be more effective in patients with a high KPS score. As the inclusion of only patients with a KPS score of ≥ 70 limits the interpretation of the current results, results from future real-world studies that include patients with a KPS score of < 70, eg, the ongoing ROSETTA study,^27^ might facilitate further evaluation.

Further studies are expected to evaluate efficacy and safety of tirabrutinib on a larger scale and with a longer follow-up period. In addition, combining tirabrutinib administration with existing treatment options such as HD-MTX-based therapy or maintenance therapy targeting patients who responded to existing treatment might further enhance the effectiveness of treatment. Additional indications of tirabrutinib in newly diagnosed PCNSL and other B-cell lymphoma types are also eagerly anticipated. In that regard, the phase 2 PROSPECT study is ongoing in the United States with an objective to assess the efficacy of tirabrutinib plus MTX-based therapy in newly diagnosed PCNSL, as well as that of tirabrutinib monotherapy in relapsed/refractory PCNSL.^13^ Results from the study are awaited with great interest.

## Limitations

This study had several limitations. For one, the number of patients in the administration groups were limited and different (320 mg, *n* = 20; 480 mg, *n* = 7; 480 mg fasted, and *n* = 17); thus, the efficacy and safety data may required further confirmation in a larger population. Next, as a single-arm design was used, the current study did not include a control arm. Therefore, superiority or inferiority of tirabrutinib to other treatment options could not be assessed. Also, the current analyses (except for the OS analysis) only included patients who were able to continue treatment, which might have caused a selection bias. Additionally, the genetic testing was performed using tumor tissues collected at the time of initial diagnosis. Thus, it is uncertain whether the results properly reflect the status of tumors at the initiation of study treatment. Finally, because the study included only Japanese patients, further studies in global populations (currently ongoing) might be needed to confirm the effectiveness and safety of tirabrutinib in different ethnicities or regions.

## Conclusion

The 3-year follow-up results demonstrated the long-term clinical benefit of tirabrutinib in patients with relapsed/refractory PCNSL, including a subset of patients with a deep and durable response, while maintaining the KPS and QoL scores. The safety profile during the extended follow-up was consistent with the previous results.

## Supplementary Material

vdae037_suppl_Supplementary_Data
