# Supplementary material for: Three-year follow-up analysis of phase 1/2 study on tirabrutinib in patients with relapsed or refractory primary central nervous system lymphoma
Source: Neurooncol Adv. 2024 Apr 22;6(1):vdae037. doi: 10.1093/noajnl/vdae037 (PMC11059299; doi:10.1093/noajnl/vdae037)
Supplement: vdae037_suppl_Supplementary_Data [file vdae037_suppl_supplementary_data.pdf]

# Supplementary data

**Three-year follow-up analysis of phase I/II study on tirabrutinib in patients with relapsed or refractory primary central nervous system lymphoma**

Hajime Yonezawa, MD, PhD, Yoshitaka Narita, MD, PhD, Motoo Nagane, MD, PhD, Kazuhiko Mishima, MD, PhD, Yasuhito Terui, MD, PhD, Yoshiki Arakawa, MD, PhD, Katsunori Asai MD, PhD, Noriko Fukuhara, MD, PhD, Kazuhiko Sugiyama, MD, PhD, Naoki Shinojima, MD, PhD, Arata Aoi, Ryo Nishikawa, MD, PhD

**Supplementary Table S1.** Exposure and disposition

**Supplementary Table S2.** Tumor responses as assessed by the central independent review committee and duration of response

**Supplementary Table S3.** Progression-free survival and overall survival in all patients and each dosage group

**Supplementary Table S4.** OS in patients with subsequent HD-MTX-based therapy or radiotherapy

**Supplementary Table S5.** Subgroup analysis of PFS at 12 months according to baseline patient characteristics

**Supplementary Table S6.** Outcome of treatment-related adverse events of special interest and treatment status of tirabrutinib in overall population

**Supplementary Figure S1.** Timeline of the study

**Supplementary Figure S2.** Subsequent therapy and survival outcomes

**Supplementary Figure S3.** Subsequent therapy and survival outcomes of those who received HD-MTX-based therapy and those who received radiotherapy

**Supplementary Figure S4.** Subgroup analysis of ORR by patient characteristics.

**Supplementary Figure S5.** Time to first onset of treatment-related AEs of special interest

**Supplementary Figure S6.** KPS in all patients and each dose group

**Supplementary Figure S7.** QLQ-BN20 in all patients and each dose group

**Supplementary Figure S8.** QLQ-C30 in all patients and each dose group

**Supplementary Figure S9.** EQ-5D-3L scores in all patients and each dose group

**Supplementary Table S1. Exposure and disposition**

| | **All**<br>**n = 44** | **320 mg**<br>**n = 20** | **480 mg**<br>**n = 7** | **480 mg fasted**<br>**n = 17** |
|---|---|---|---|---|
| Median duration of treatment, months (range) | 2.7 (0.8–46.9) | 2.3 (0.9–46.9) | 11.1 (0.8–29.6) | 7.4 (0.9–39.6) |
| Treatment completion, n (%) | 5 (11.4) | 1 (5.0) | 0 | 4 (23.5) |
| Treatment discontinuation, n (%) | 39 (88.6) | 19 (95.0) | 7 (100.0) | 13 (76.5) |
| Reason for treatment discontinuation, n (%) | | | | |
| Disease progression | 31 (70.5) | 14 (70.0) | 5 (71.4) | 12 (70.6) |
| Adverse event | 4 (9.1) | 2 (10.0) | 2 (28.6) | 0 |
| Other* | 4 (9.1) | 3 (15.0) | 0 | 1 (5.9) |

*The investigator or sub-investigator decided that it was inappropriate to continue the study treatment. q.d., once daily.

**Supplementary Table S2. Tumor responses as assessed by the central independent review committee and duration of response**

| | **All**<br>**n = 44** | **320 mg**<br>**n = 20** | **480 mg**<br>**n = 7** | **480 mg fasted**<br>**n = 17** |
|---|---|---|---|---|
| ORR (CR + CRu + PR), n (%) | 28 (63.6) | 12 (60.0) | 7 (100.0) | 9 (52.9) |
| 95% CI, % | 47.8–77.6 | 36.1–80.9 | 59.0–100.0 | 27.8–77.0 |
| CR rate (CR + CRu), n (%) | 16 (36.4) | 5 (25.0) | 4 (57.1) | 7 (41.2) |
| 95% CI, % | 22.4–52.2 | 8.7–49.1 | 18.4–90.1 | 18.4–67.1 |
| BOR, n (%) | | | | |
| CR | 9 (20.5) | 3 (15.0) | 1 (14.3) | 5 (29.4) |
| CRu | 7 (15.9) | 2 (10.0) | 3 (42.9) | 2 (11.8) |
| PR | 12 (27.3) | 7 (35.0) | 3 (42.9) | 2 (11.8) |
| SD | 7 (15.9) | 4 (20.0) | 0 | 3 (17.6) |
| PD | 9 (20.5) | 4 (20.0) | 0 | 5 (29.4) |
| Median TTR, months (range)* | 0.9 (0.3–1.2) | 0.9 (0.9–1.2) | 0.9 (0.3–1.0) | 0.9 (0.8–1.0) |
| Median DOR, months (95% CI)* | 9.2 (1.7–17.2) | 3.7 (0.9–19.4) | 10.2 (0.6–21.2) | 12.1 (0.9–NR) |
| 12-month DOR rate, % (95% CI)* | 46.4 (27.6–63.3) | 41.7 (15.2–66.5) | 42.9 (9.8–73.4) | 55.6 (20.4–80.5) |
| 24-month DOR rate, % (95% CI)* | 23.8 (10.0–40.9) | 20.8 (3.5–47.9) | 14.3 (0.7.–46.5) | 33.3 (7.8–62.3) |
| 36-month DOR rate, % (95% CI)* | 19.8 (7.4–36.5) | 20.8 (3.5–47.9) | 0 | 33.3 (7.8–62.3) |

*Among patients who achieved CR, CRu, or PR (n = 28, 12, 7, and 9 for all patients, 320 mg, 480 mg, and 480 mg fasted groups, respectively). BOR, best overall response; CI, confidence interval; CR, complete response; CRu, unconfirmed complete response; DOR, duration of response; NR, not reached; ORR, overall response rate; PD, progressive disease; PR, partial response; q.d., once daily; SD, stable disease; TTR, time to response

**Supplementary Table S3. Progression-free survival and overall survival in all patients and each dosage group**

| | All<br>n = 44 | 320 mg<br>n = 20 | 480 mg<br>n = 7 | 480 mg fasted<br>n = 17 |
|---|---|---|---|---|
| Median PFS, months (95% CI) | 2.9 (1.8–11.1) | 2.1 (1.8–18.2) | 11.1 (1.4–22.0) | 5.8 (1.0–13.0) |
| 12-month PFS rate, % (95% CI) | 32.5 (18.9–46.9) | 29.4 (11.0–50.7) | 42.9 (9.8–73.4) | 31.7 (11.6–54.1) |
| 24-month PFS rate, % (95% CI) | 16.7 (7.0–29.9) | 14.7 (2.7–36.2) | 14.3 (0.7–46.5) | 19.0 (4.7–40.6) |
| 36-month PFS rate, % (95% CI) | 13.9 (5.3–26.7) | 14.7 (2.7–36.2) | 0 | 19.0 (4.7–40.6) |
| Median OS, months (95% CI) | NR (21.0–NR) | 37.9 (11.2–NR) | NR (1.4–NR) | NR (5.5–NR) |
| 12-month OS rate, % (95% CI) | 72.7 (57.0–83.5) | 75.0 (50.0–88.8) | 85.7 (33.4–97.9) | 64.7 (37.7–82.3) |
| 24-month OS rate, % (95% CI) | 61.4 (45.4–74.0) | 60.0 (35.7–77.6) | 85.7 (33.4–97.9) | 52.9 (27.6–73.0) |
| 36-month OS rate, % (95% CI) | 56.7 (40.9–69.8) | 55.0 (31.3–73.5) | 71.4 (25.8–92.0) | 52.9 (27.6–73.0) |

CI, confidence interval; NR, not reached; PFS, progression-free survival; OS, overall survival.

**Supplementary Table S4. OS in patients with subsequent HD-MTX-based therapy or radiotherapy**

| | **Subsequent HD-MTX n = 18** | **Subsequent WBRT n = 10** |
|---|---|---|
| Median OS, months (95% CI) | NR (21.0–NR) | 29.0 (5.5–NR) |
| 12-month OS, % (95% CI) | 83.3 (56.8–94.3) | 70.0 (32.9–89.2) |
| 24-month OS, % (95% CI) | 61.1 (35.3–79.2) | 60.0 (25.3–82.7) |
| 36-month OS, % (95% CI) | 61.1 (35.3–79.2) | 40.0 (12.3–67.0) |

CI, confidence interval; HD-MTX, high-dose methotrexate NR, not reached; OS, over survival; WBRT, whole-brain radiotherapy.

**Supplementary Table S5. Subgroup analysis of PFS at 12 months according to baseline patient characteristics**

| | | | Univariate analysis | | | Multivariate analysis | | |
|---|---|---|---|---|---|---|---|---|
| **Factor** | **n** | **PFS events at 12 months, n (%)** | **Odds ratio** | **95% CI** | ***P*-value** | **Odds ratio** | **95% CI** | ***P*-value** |
| **Age, y** | | | | | | | | |
| <65 | 22 | 16 (72.7) | Reference | | | | | |
| ≥65 | 19 | 12 (63.2) | 0.643 | 0.171–2.413 | 0.5126 | | | |
| **KPS** | | | | | | | | |
| 70–80 | 21 | 18 (85.7) | 5.999 | 1.333–26.996 | 0.0196* | 5.993 | 1.202–29.876 | 0.0289* |
| 90–100 | 20 | 10 (50.0) | Reference | | | Reference | | |
| **Number of previous lines of treatment** | | | | | | | | |
| 1 | 17 | 9 (52.9) | Reference | | | Reference | | |
| ≥2 | 24 | 19 (79.2) | 3.378 | 0.858–13.296 | 0.0817 | 3.292 | 0.701–15.451 | 0.1310 |
| **Previous rituximab** | | | | | | | | |
| Yes | 23 | 16 (69.6) | 1.143 | 0.305–4.289 | 0.8431 | | | |
| No | 18 | 12 (66.7) | Reference | | | | | |
| **Previous WBRT** | | | | | | | | |
| Yes | 27 | 21 (77.8) | 3.500 | 0.875–13.995 | 0.0764 | 3.051 | 0.634–14.671 | 0.1639 |
| No | 14 | 7 (50.0) | Reference | | | Reference | | |
| **Previous HCT-ASCT** | | | | | | | | |
| Yes | 5 | 4 (80.0) | 2.000 | 0.201–19.914 | 0.5544 | | | |
| No | 36 | 24 (66.7) | Reference | | | | | |
| **Disease status** | | | | | | | | |
| Relapse | 32 | 21 (65.6) | Reference | | | | | |
| Refractory | 7 | 6 (85.7) | 3.143 | 0.335–29.493 | 0.2603 | | | |
| Unknown | 2 | 1 (50.0) | 0.524 | 0.030–9.203 | 0.4241 | | | |
| **CNS involvement** | | | | | | | | |
| CSF | | | | | | | | |
| Positive | 9 | 7 (77.8) | 1.833 | 0.324–10.367 | 0.4929 | | | |
| Negative | 32 | 21 (65.6) | Reference | | | | | |
| IOL | | | | | | | | |
| Positive | 3 | 2 (66.7) | 0.870 | 0.070–10.728 | 0.9553 | | | |
| Minor RPE abnormality | 5 | 3 (60.0) | 0.652 | 0.094–4.525 | 0.7485 | | | |
| Negative | 33 | 23 (69.7) | Reference | | | | | |
| **GCB subtype** | | | | | | | | |
| GCB | 13 | 8 (61.5) | 0.640 | 0.160–2.559 | 0.5279 | | | |
| Non-GCB | 28 | 20 (71.4) | Reference | | | | | |
| **Gene mutation (*CARD11*)** | | | | | | | | |
| Yes | 16 | 10 (62.5) | 0.648 | 0.170–2.467 | 0.5249 | | | |
| No | 25 | 18 (72.0) | Reference | | | | | |
| **Gene mutation (*CD79B*)** | | | | | | | | |
| Yes | 15 | 9 (60.0) | 0.553 | 0.143–2.128 | 0.3886 | | | |
| No | 26 | 19 (73.1) | Reference | | | | | |
| **Gene mutation (*MYD88*)** | | | | | | | | |
| Yes | 29 | 21 (72.4) | 1.875 | 0.459–7.659 | 0.3812 | | | |
| No | 12 | 7 (58.3) | Reference | | | | | |
| **SPD at target lesion, $mm^2$** | | | | | | | | |
| <400 | 23 | 15 (65.2) | 0.721 | 0.189–2.759 | 0.6330 | | | |
| ≥400 | 18 | 13 (72.2) | Reference | | | | | |

*$P$ <0.05. After excluding 3 patients who were censored at 12 months without PD or death, 41 patients were included in this analysis. The variables for the multivariate analysis of PFS (KPS, number of previous lines of treatment, and previous WBRT) were chosen with the step-wise method. CI, confidence interval; CNS, central nervous system; CSF, cerebrospinal fluid; GCB, germinal center B-cell-like; IOL, intraocular lymphoma; HCT-ASCT, high-dose chemotherapy followed by autologous stem cell transplantation; PD, progressive disease; PFS, progression-free survival; RPE, retinal pigment epithelial; SPD, sum of the products of the greatest diameters; WBRT, whole-brain radiotherapy.

**Supplementary Table S6. Outcome of treatment-related adverse events of special interest and treatment status of tirabrutinib in overall population**

| TRAEs of special interest | Onset, n (%) | Median time to onset, days (range) | Recovered or recovering, n (%)* | Median time to recovered or recovering, days (range) | Tirabrutinib treatment, n (%)* | | Medical intervention for the TRAEs, n (%)* |
|---|---|---|---|---|---|---|---|
| Skin | 24 (54.5) | 13.0 (1–121) | 20 (83.3) | 35.5 (6–635) | Dose not changed | 15 (62.5) | 20 (83.3) |
| | | | | | Dose reduced | 1 (4.2) | |
| | | | | | Drug interrupted | 6 (25.0) | |
| | | | | | Drug withdrawn | 2 (8.3) | |
| | | | | | Unknown | 0 | |
| Cytopenia | 19 (43.2) | 15.0 (2–813) | 15 (78.9) | 30.0 (14–682) | Dose not changed | 17 (89.5) | 1 (5.3) |
| | | | | | Dose reduced | 0 | |
| | | | | | Drug interrupted | 2 (10.5) | |
| | | | | | Drug withdrawn | 0 | |
| | | | | | Unknown | 0 | |
| Infection | 7 (15.9) | 130.0 (32–346) | 5 (71.4) | 18.0 (6–25) | Dose not changed | 1 (14.3) | 7 (100) |
| | | | | | Dose reduced | 0 | |
| | | | | | Drug interrupted | 4 (57.1) | |
| | | | | | Drug withdrawn | 1 (14.3) | |
| | | | | | Unknown | 1 (14.3) | |
| Haemorrhage | 3 (6.8) | 29.0 (10–109) | 3 (100) | 48.0 (18–190) | Dose not changed | 2 (66.7) | 0 |
| | | | | | Dose reduced | 0 | |
| | | | | | Drug interrupted | 1 (33.3) | |
| | | | | | Drug withdrawn | 0 | |
| | | | | | Unknown | 0 | |
| Diarrhoea | 1 (2.3) | 2.0 (2–2) | 1 (100) | 2.0 (2–2) | Dose not changed | 1 (100) | 0 |
| | | | | | Dose reduced | 0 | |
| | | | | | Drug interrupted | 0 | |
| | | | | | Drug withdrawn | 0 | |
| | | | | | Unknown | 0 | |

*Among those who developed each TRAE. TRAE, treatment-related adverse events.

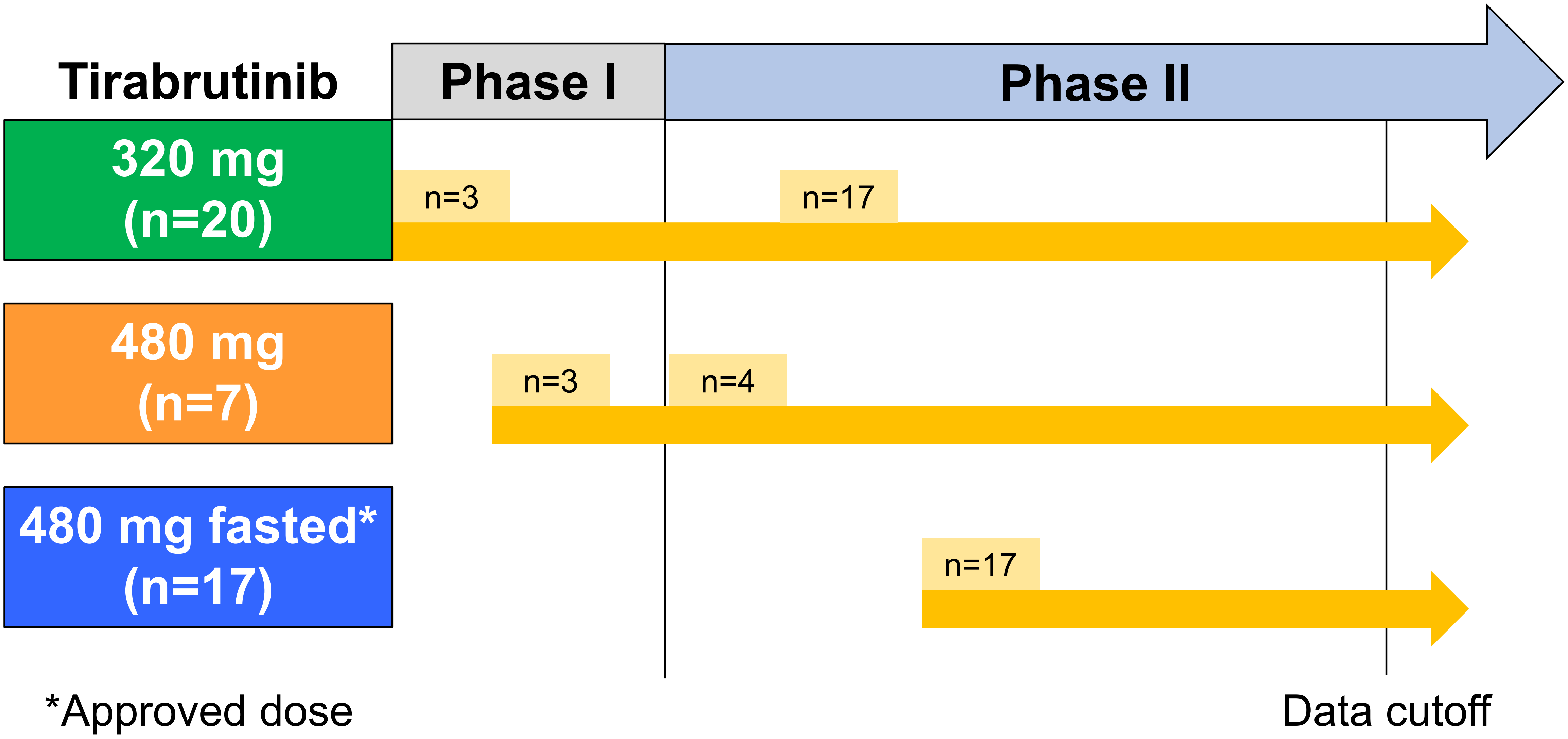


**Supplementary Figure S1. Timeline of the study**

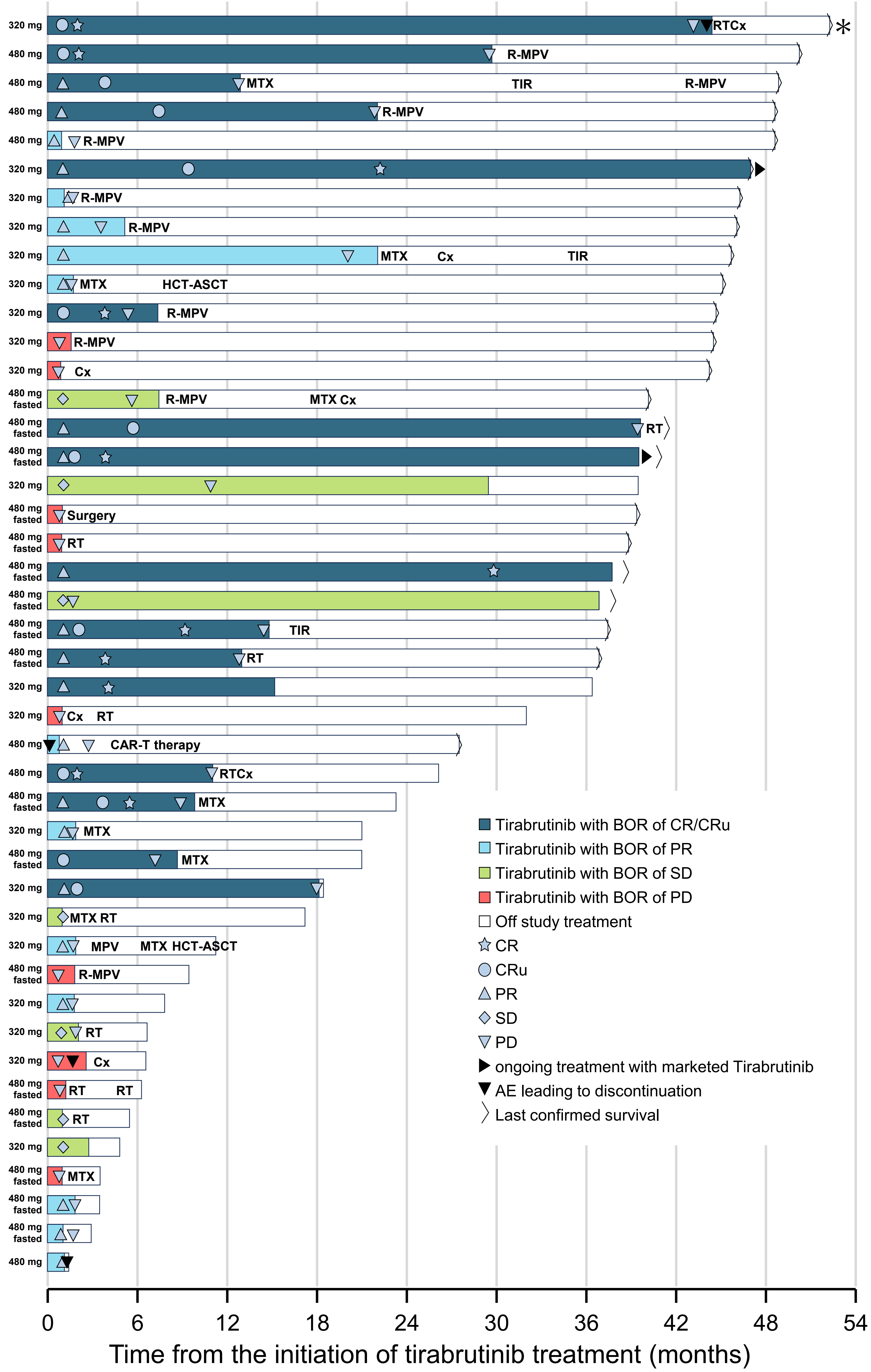


**Supplementary Figure S2. Subsequent therapy and survival outcomes**

BOR is per central assessment and duration of treatment is per investigators' assessment. *The patient developed an adverse event on a date on which he or she had centrally confirmed PD. AE, adverse event; BOR, best overall response; CR, complete response; CRu, unconfirmed complete response; Cx, chemotherapy except HD-MTX-based therapy; HCT-ASCT, high-dose chemotherapy followed by autologous stem cell transplantation; HD-MTX, high-dose methotrexate; MTX, HD-MTX-based therapy excluding R-MPV and MPV; MPV, methotrexate, procarbazine, and vincristine; R-MPV, rituximab plus MPV; SD, stable disease; TIR, Tirabrutinib re-challenged; PD, progressive disease; PR, partial response; RT, radiotherapy.

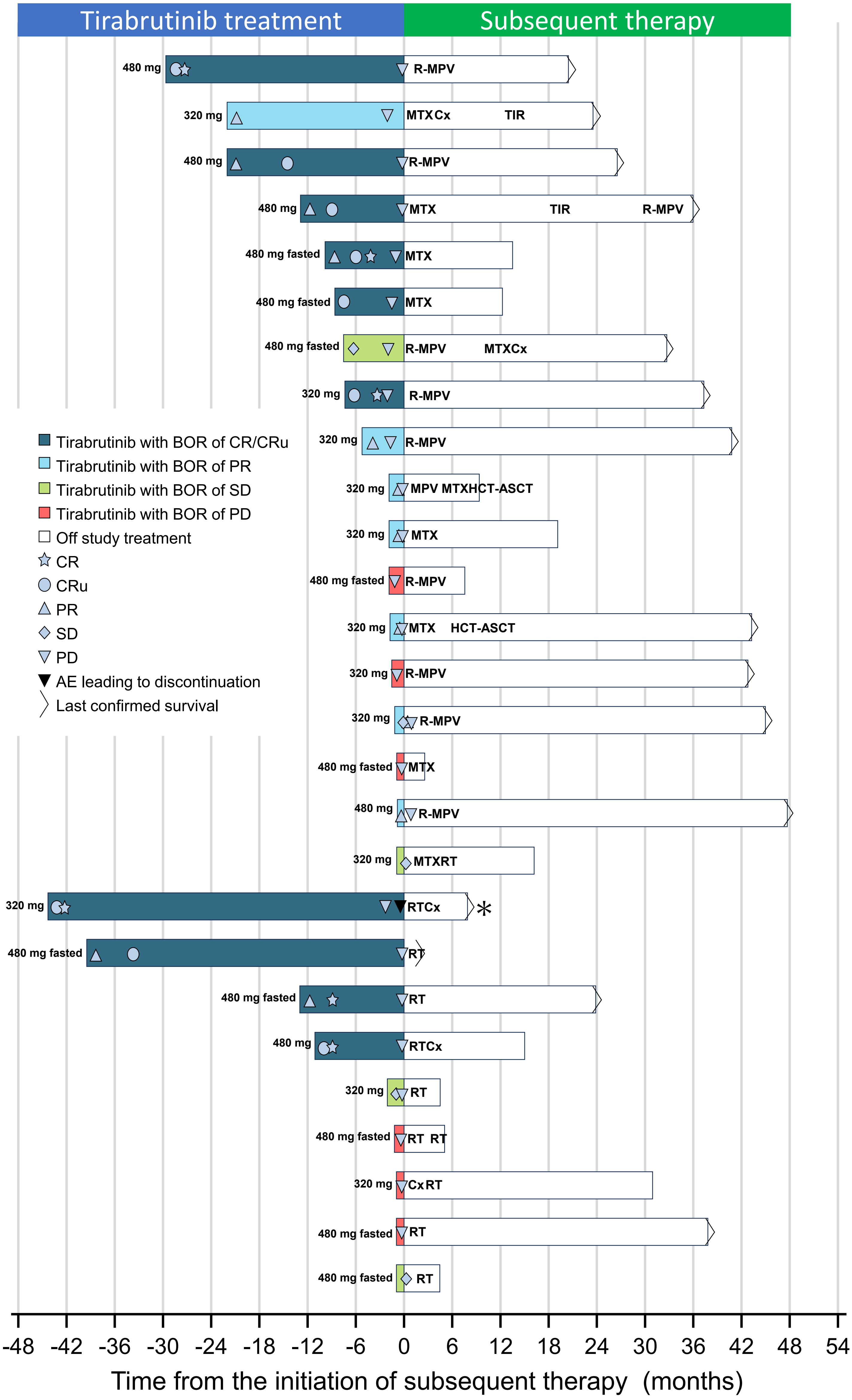


**Supplementary Figure S3. Subsequent therapy and survival outcomes of those who received HD-MTX-based therapy and those who received radiotherapy**

BOR is per central assessment and duration of treatment is per investigators' assessment. *The patient developed an adverse event on a date on which he or she had centrally confirmed PD. AE, adverse event; BOR, best overall response; CR, complete response; CRu, unconfirmed complete response; Cx, chemotherapy except HD-MTX-based therapy; HCT-ASCT, high-dose chemotherapy followed by autologous stem cell transplantation; HD-MTX, high-dose methotrexate; MTX, HD-MTX-based therapy excluding R-MPV and MPV; MPV, methotrexate, procarbazine, and vincristine; R-MPV, rituximab plus MPV; SD, stable disease; TIR, Tirabrutinib re-challenged; PD, progressive disease; PR, partial response; RT, radiotherapy.

**Supplementary Figure S4. See the figure legend on the next page**

| | n | ORR (%) | [95% CI]* |
|---|---|---|---|
| **All** | **44** | **28 ( 63.6)** | **[ 47.8, 77.6 ]** |
| **Sex** | | | |
| Male | 24 | 14 ( 58.3) | [ 36.6, 77.9 ] |
| Female | 20 | 14 ( 70.0) | [ 45.7, 88.1 ] |
| **Age (years)** | | | |
| <65 | 24 | 15 ( 62.5) | [ 40.6, 81.2 ] |
| 65 to <75 | 14 | 9 ( 64.3) | [ 35.1, 87.2 ] |
| ≥75 | 6 | 4 ( 66.7) | [ 22.3, 95.7 ] |
| **Time from the diagnosis of PCNSL to enrollment (initial onset) (months)** | | | |
| <35 | 23 | 14 ( 60.9) | [ 38.5, 80.3 ] |
| ≥35 | 15 | 10 ( 66.7) | [ 38.4, 88.2 ] |
| **Time from the diagnosis of PCNSL to enrollment (last onset) (months)** | | | |
| <0.5 | 23 | 14 ( 60.9) | [ 38.5, 80.3 ] |
| ≥0.5 | 21 | 14 ( 66.7) | [ 43.0, 85.4 ] |
| **Recurrent or refractory after last treatment for PCNSL** | | | |
| Recurrent | 33 | 23 ( 69.7) | [ 51.3, 84.4 ] |
| Refractory | 9 | 3 ( 33.3) | [ 7.5, 70.1 ] |
| **Recurrent or refractory after last methotrexate therapy for PCNSL** | | | |
| Recurrent | 33 | 24 ( 72.7) | [ 54.5, 86.7 ] |
| Refractory | 10 | 3 ( 30.0) | [ 6.7, 65.2 ] |
| **Recurrent or refractory after last rituximab therapy for PCNSL** | | | |
| Recurrent | 20 | 13 ( 65.0) | [ 40.8, 84.6 ] |
| Refractory | 5 | 1 ( 20.0) | [ 0.5, 71.6 ] |
| **Number of previous lines of treatments** | | | |
| 1 | 18 | 13 ( 72.2) | [ 46.5, 90.3 ] |
| 2–3 | 16 | 9 ( 56.3) | [ 29.9, 80.2 ] |
| ≥4 | 10 | 6 ( 60.0) | [ 26.2, 87.8 ] |
| **Number of previous lines of chemotherapies** | | | |
| 1 | 23 | 17 ( 73.9) | [ 51.6, 89.8 ] |
| 2–3 | 15 | 9 ( 60.0) | [ 32.3, 83.7 ] |
| ≥4 | 6 | 2 ( 33.3) | [ 4.3, 77.7 ] |
| **Treatment history for PCNSL (HCT-ASCT)** | | | |
| Yes | 7 | 2 ( 28.6) | [ 3.7, 71.0 ] |
| No | 37 | 26 ( 70.3) | [ 53.0, 84.1 ] |
| **Treatment history for PCNSL (chemotherapies)** | | | |
| Yes | 44 | 28 ( 63.6) | [ 47.8, 77.6 ] |
| No | 0 | - | - |
| **Treatment history for PCNSL (WBRT)** | | | |
| Yes | 29 | 18 ( 62.1) | [ 42.3, 79.3 ] |
| No | 15 | 10 ( 66.7) | [ 38.4, 88.2 ] |
| **Treatment history for PCNSL (methotrexate)** | | | |
| Yes | 44 | 28 ( 63.6) | [ 47.8, 77.6 ] |
| No | 0 | - | - |

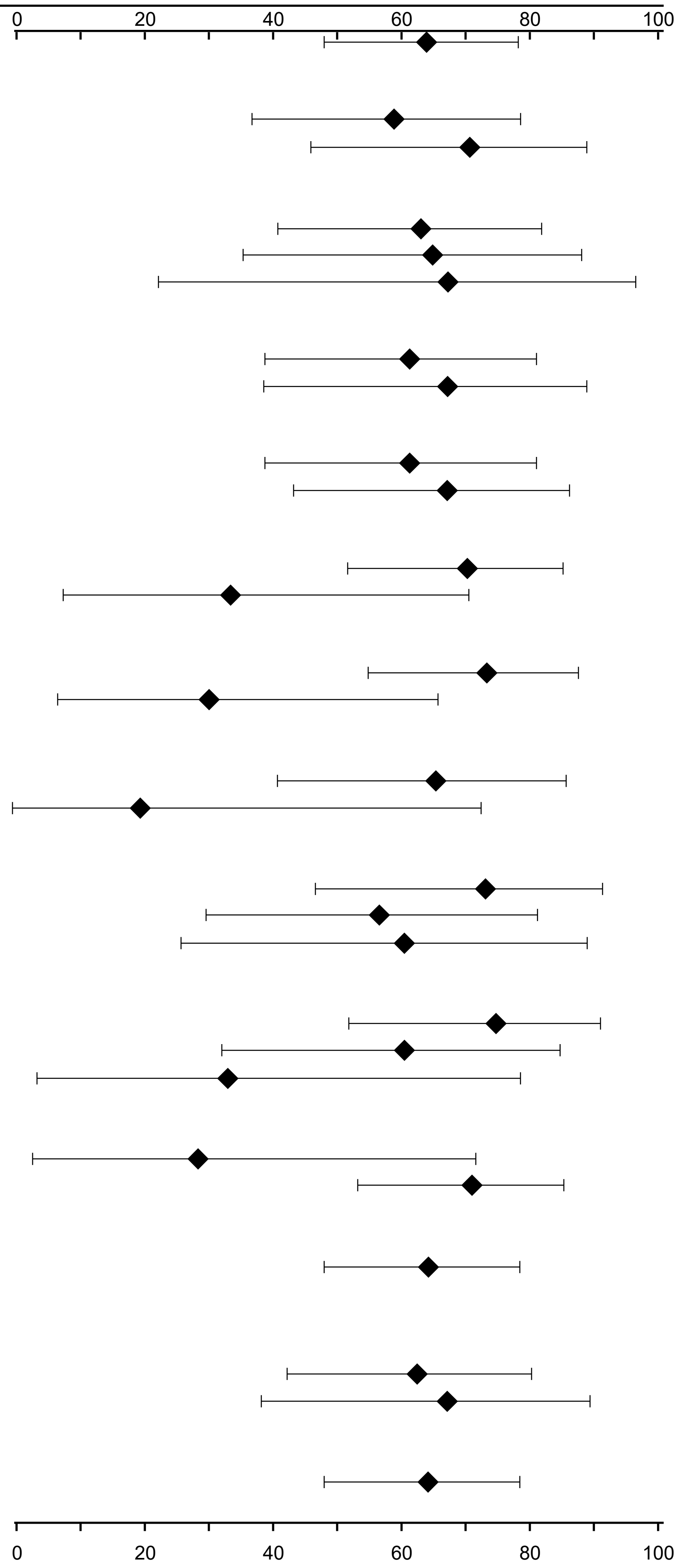

| | n | ORR (%) | [95% CI]* |
|---|---|---|---|
| **Treatment history for PCNSL (cytarabine)** | | | |
| Yes | 21 | 12 ( 57.1) | [ 34.0, 78.2 ] |
| No | 23 | 16 ( 69.6) | [ 47.1, 86.8 ] |
| **Treatment history for PCNSL (rituximab)** | | | |
| Yes | 26 | 14 ( 53.8) | [ 33.4, 73.4 ] |
| No | 18 | 14 ( 77.8) | [ 52.4, 93.6 ] |
| **Prognostic factors (Recursive partitioning analysis scoring system)** | | | |
| Class 1 | 11 | 7 ( 63.6) | [ 30.8, 89.1 ] |
| Class 2 | 33 | 21 ( 63.6) | [ 45.1, 79.6 ] |
| Class 3 | 0 | - | - |
| **Gene mutation (CARD11)** | | | |
| Yes | 17 | 10 ( 58.8) | [ 32.9, 81.6 ] |
| No | 27 | 18 ( 66.7) | [ 46.0, 83.5 ] |
| **Gene mutation (CD79A)** | | | |
| Yes | 2 | 1 ( 50.0) | [ 1.3, 98.7 ] |
| No | 42 | 27 ( 64.3) | [ 48.0, 78.4 ] |
| **Gene mutation (CD79B)** | | | |
| Yes | 18 | 9 ( 50.0) | [ 26.0, 74.0 ] |
| No | 26 | 19 ( 73.1) | [ 52.2, 88.4 ] |
| **Gene mutation (MYD88)** | | | |
| Yes | 32 | 19 ( 59.4) | [ 40.6, 76.3 ] |
| No | 12 | 9 ( 75.0) | [ 42.8, 94.5 ] |
| **Gene mutation (CD79A/B)**** | | | |
| Yes | 18 | 9 ( 50.0) | [ 26.0, 74.0 ] |
| No | 26 | 19 ( 73.1) | [ 52.2, 88.4 ] |
| **Gene mutation (CD79B and MYD88)** | | | |
| Yes-Yes | 15 | 6 ( 40.0) | [ 16.3, 67.7 ] |
| Yes-No | 3 | 3 (100.0) | [ 29.2, 100.0 ] |
| No-Yes | 17 | 13 ( 76.5) | [ 50.1, 93.2 ] |
| No-No | 9 | 6 ( 66.7) | [ 29.9, 92.5 ] |
| **PCNSL subtype** | | | |
| GCB | 13 | 11 ( 84.6) | [ 54.6, 98.1 ] |
| Non-GCB | 31 | 17 ( 54.8) | [ 36.0, 72.7 ] |
| Unknown | 0 | - | - |
| **SPD at target lesion ($mm^2$) (central review)** | | | |
| <400 | 23 | 19 ( 82.6) | [ 61.2, 95.0 ] |
| ≥400 | 21 | 9 ( 42.9) | [ 21.8, 66.0 ] |
| **Karnofsky Performance Status** | | | |
| ≤80 | 24 | 15 ( 62.5) | [ 40.6, 81.2 ] |
| 90–100 | 20 | 13 ( 65.0) | [ 40.8, 84.6 ] |

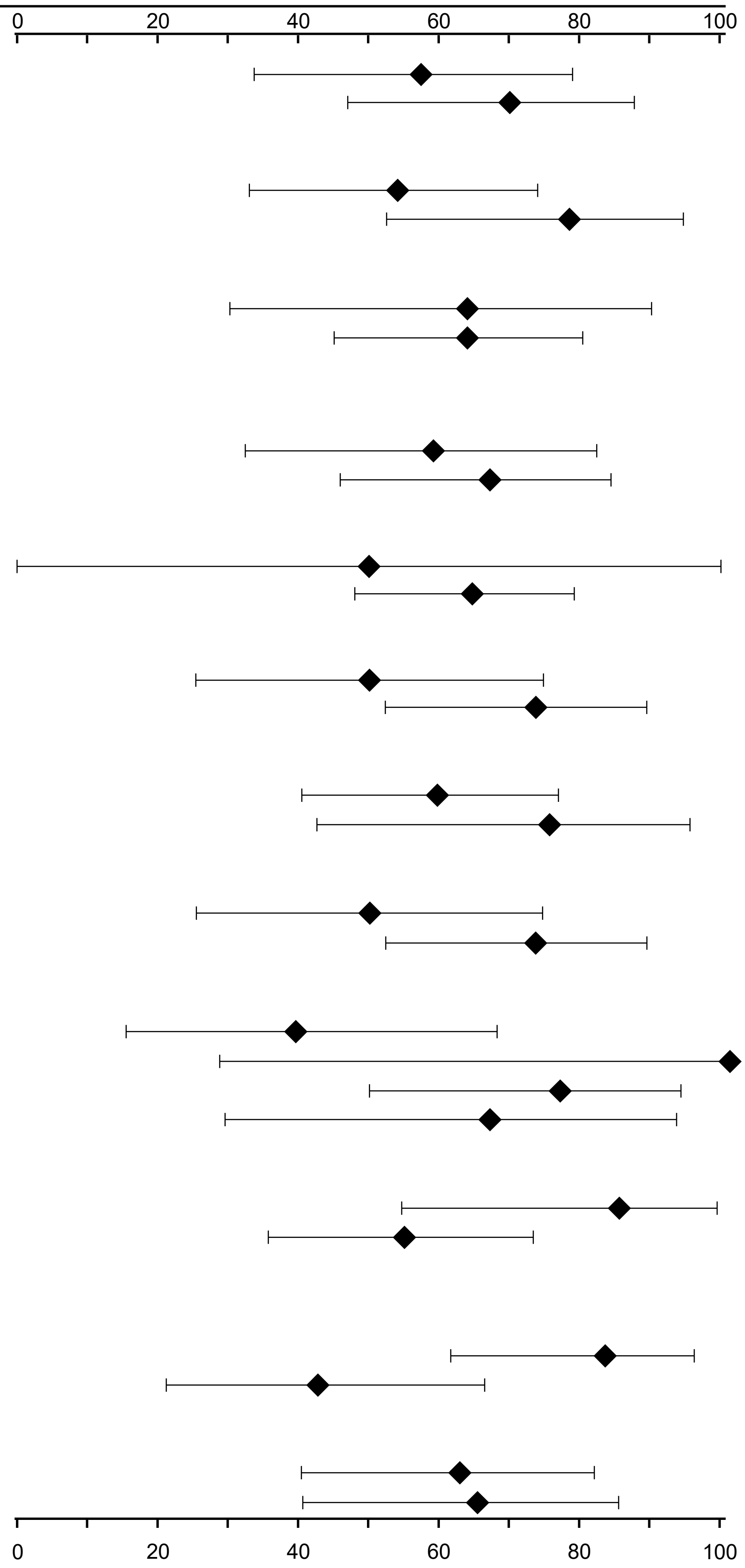


**Supplementary Figure 4. Subgroup analysis of ORR by patient characteristics.**
The best overall response was assessed according to the International PCNSL Collaborative Group (IPCG) criteria.
*The two-sided 95% confidence interval was calculated using the Clopper-Pearson method.
** "Yes" indicates that the patient had mutation in CD79A or CD79B. "No" indicates that the patient had no mutation in either gene.
CI, confidence interval; GCB, germinal center B-cell-like; HCT-ASCT, high-dose chemotherapy followed by autologous stem cell transplantation; ORR, overall response ratio; PCNSL, primary central nervous system lymphoma; SPD sum of the products of the greatest diameters; WBRT, whole-brain radiotherapy.

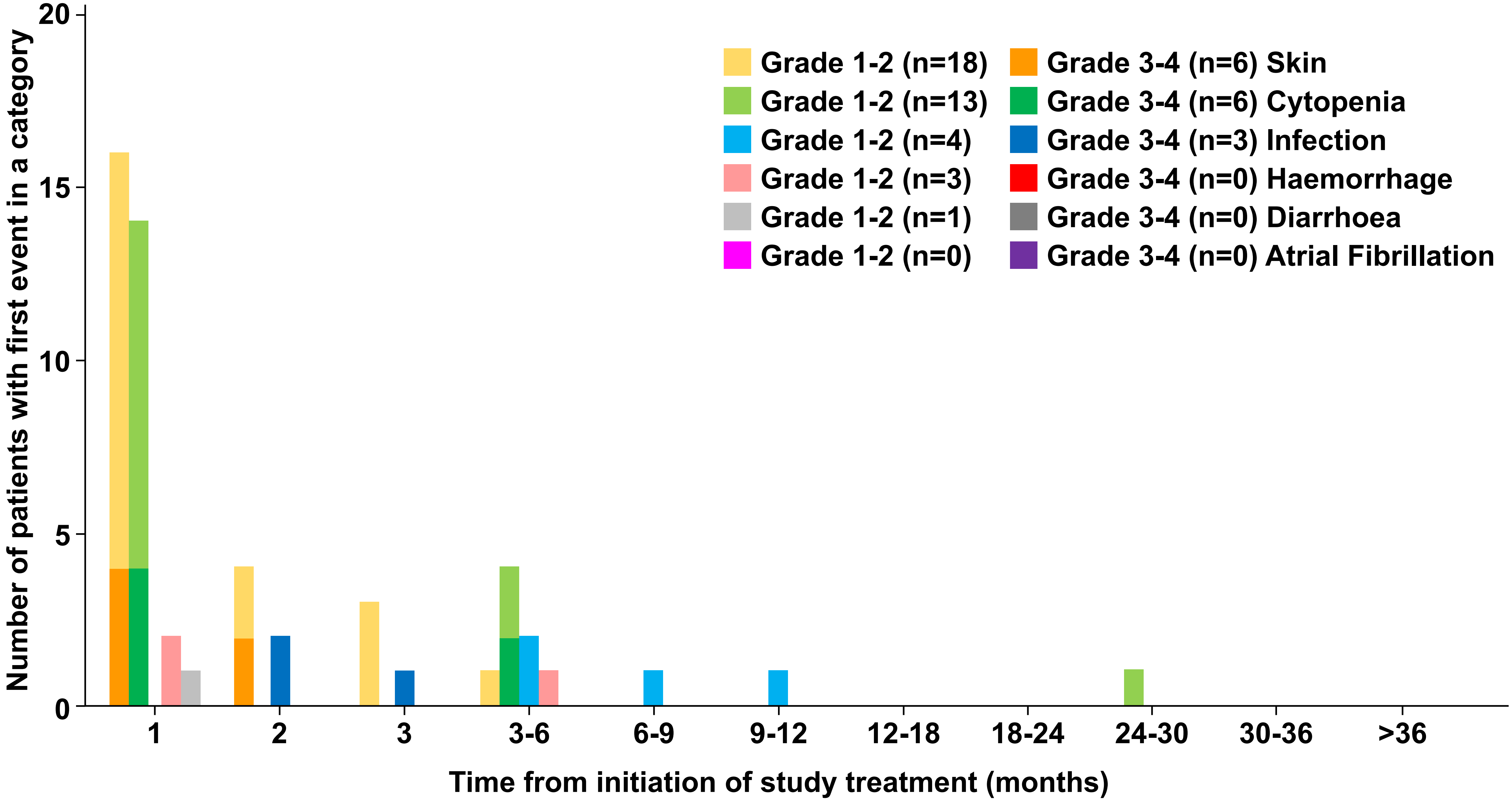


**Supplementary Figure S5. Time to first onset of treatment-related AEs of special interest**
AE, adverse events.

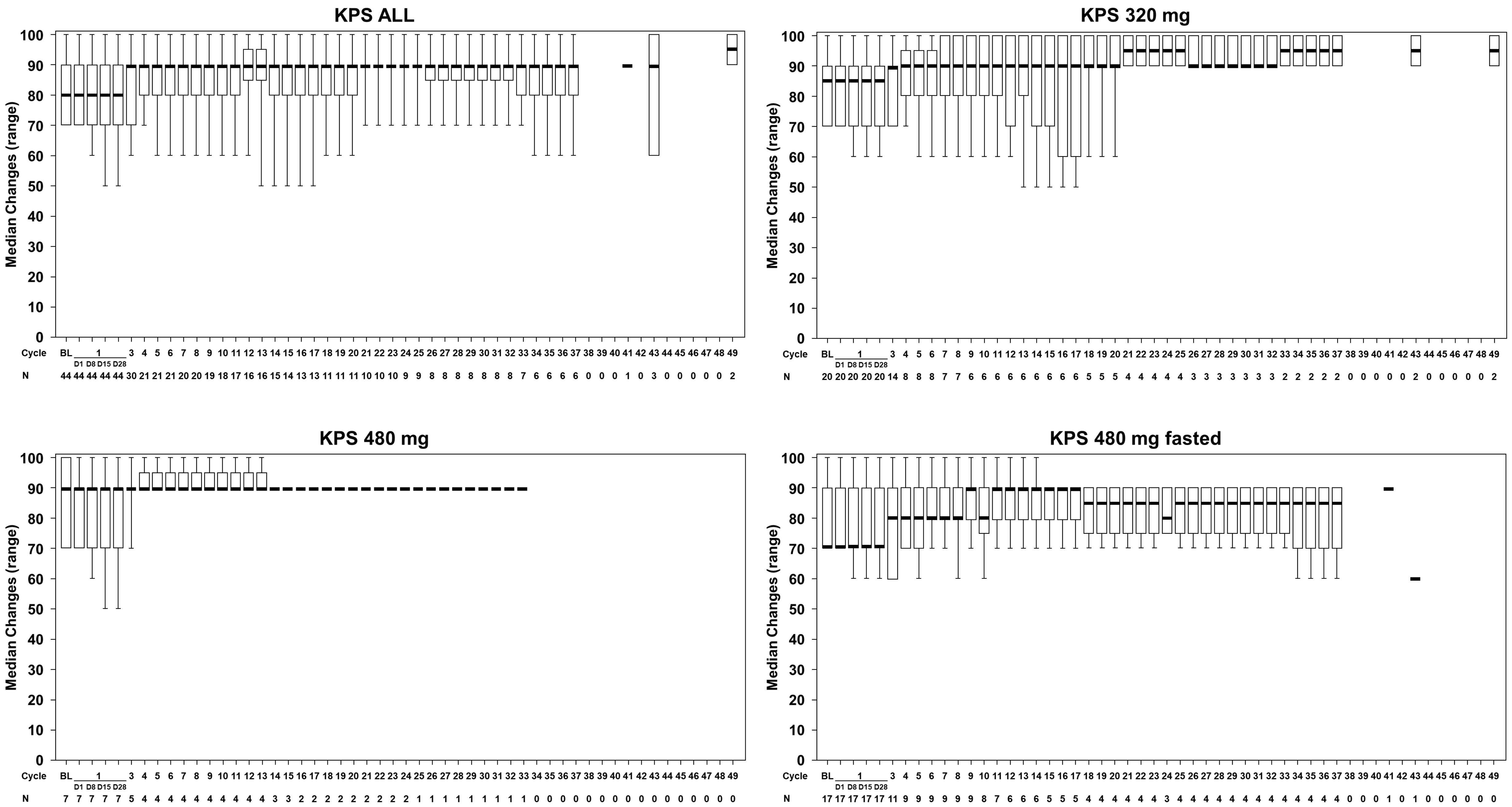


**Supplementary Figure S6. KPS in all patients and each dose group**

Boxplot diagrams for the KPS scores in each cycle of treatment. The boxes, thick line inside the boxes, and whiskers indicate the interquartile ranges, medians, and ranges, respectively. BL, baseline.

# Supplementary Figure S7. See figure legend for the later page

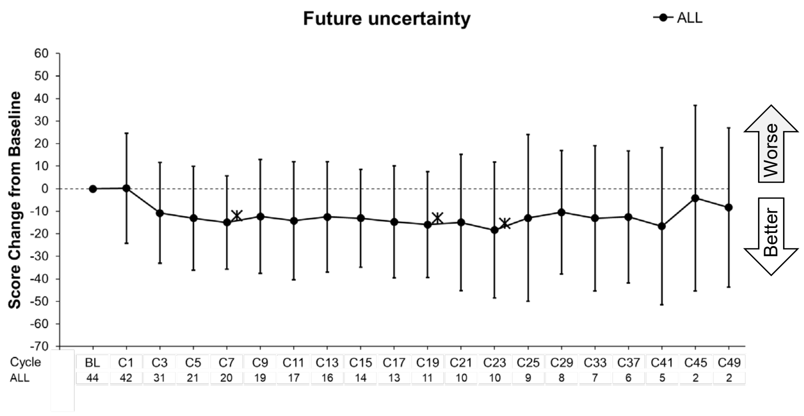


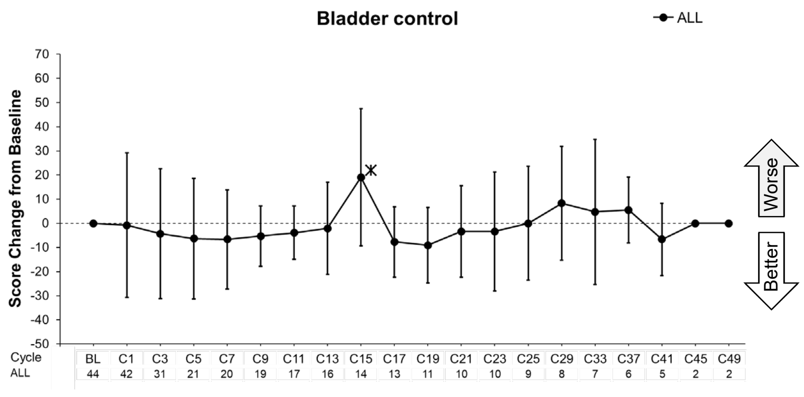


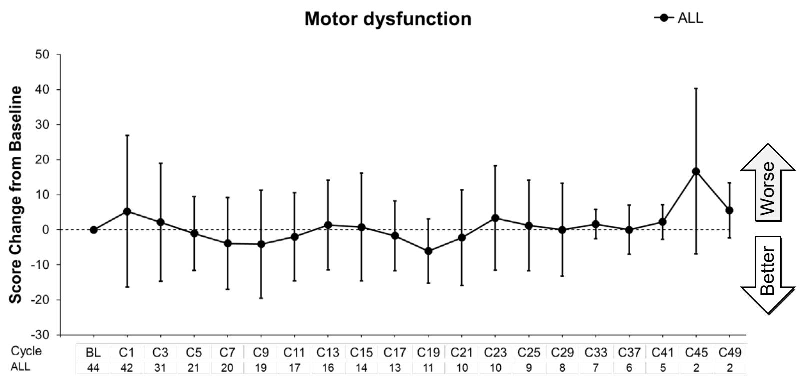


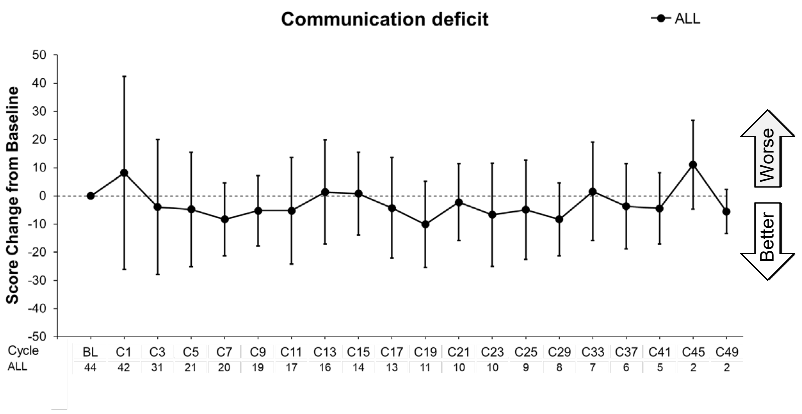


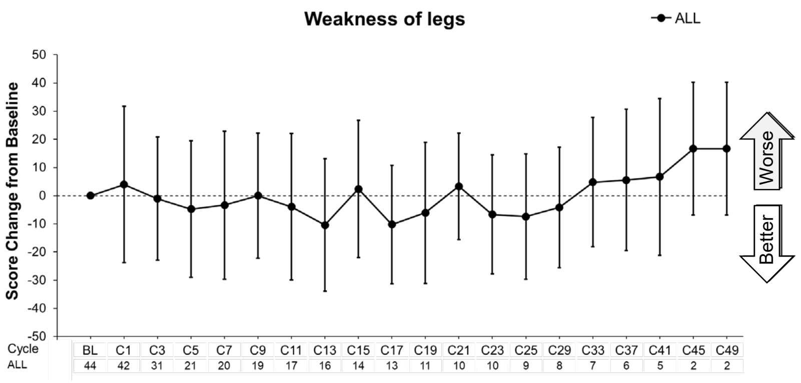

## Supplementary Figure S7. See figure legend for the later page

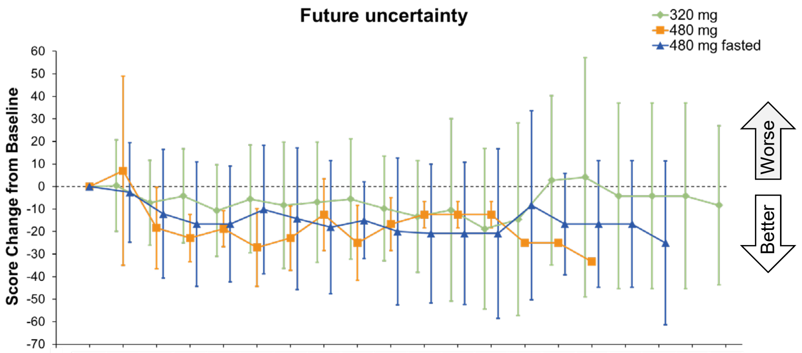


| Cycle | BL | C1 | C3 | C5 | C7 | C9 | C11 | C13 | C15 | C17 | C19 | C21 | C23 | C25 | C29 | C33 | C37 | C41 | C45 | C49 |
|---|---|---|---|---|---|---|---|---|---|---|---|---|---|---|---|---|---|---|---|---|
| 320 mg | 20 | 20 | 15 | 8 | 7 | 6 | 6 | 6 | 6 | 6 | 5 | 4 | 4 | 4 | 3 | 2 | 2 | 2 | 2 | 2 |
| 480 mg | 7 | 6 | 5 | 4 | 4 | 4 | 4 | 4 | 3 | 2 | 2 | 2 | 2 | 1 | 1 | 1 | 0 | 0 | 0 | 0 |
| 480 mg fasted | 17 | 16 | 11 | 9 | 9 | 9 | 7 | 6 | 5 | 5 | 4 | 4 | 4 | 4 | 4 | 4 | 4 | 3 | 0 | 0 |

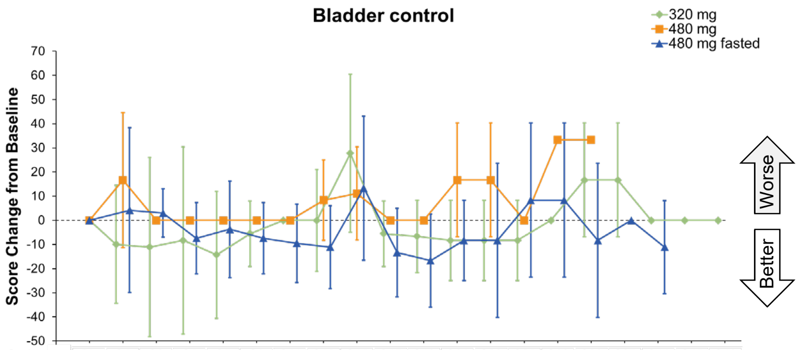


| Cycle | BL | C1 | C3 | C5 | C7 | C9 | C11 | C13 | C15 | C17 | C19 | C21 | C23 | C25 | C29 | C33 | C37 | C41 | C45 | C49 |
|---|---|---|---|---|---|---|---|---|---|---|---|---|---|---|---|---|---|---|---|---|
| 320 mg | 20 | 20 | 15 | 8 | 7 | 6 | 6 | 6 | 6 | 6 | 5 | 4 | 4 | 4 | 3 | 2 | 2 | 2 | 2 | 2 |
| 480 mg | 7 | 6 | 5 | 4 | 4 | 4 | 4 | 4 | 3 | 2 | 2 | 2 | 2 | 1 | 1 | 1 | 0 | 0 | 0 | 0 |
| 480 mg fasted | 17 | 16 | 11 | 9 | 9 | 9 | 7 | 6 | 5 | 5 | 4 | 4 | 4 | 4 | 4 | 4 | 4 | 3 | 0 | 0 |

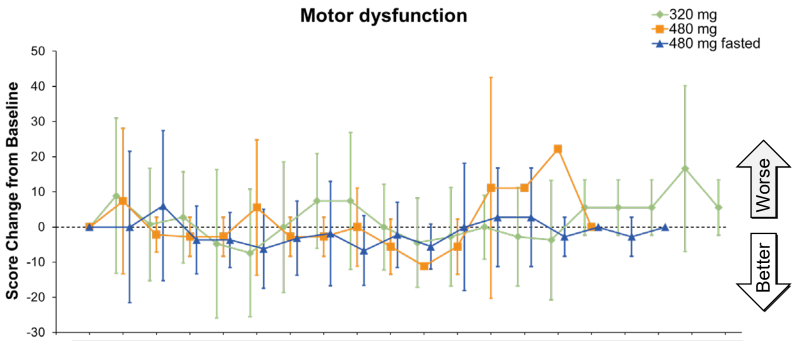


| Cycle | BL | C1 | C3 | C5 | C7 | C9 | C11 | C13 | C15 | C17 | C19 | C21 | C23 | C25 | C29 | C33 | C37 | C41 | C45 | C49 |
|---|---|---|---|---|---|---|---|---|---|---|---|---|---|---|---|---|---|---|---|---|
| 320 mg | 20 | 20 | 15 | 8 | 7 | 6 | 6 | 6 | 6 | 6 | 5 | 4 | 4 | 4 | 3 | 2 | 2 | 2 | 2 | 2 |
| 480 mg | 7 | 6 | 5 | 4 | 4 | 4 | 4 | 4 | 3 | 2 | 2 | 2 | 2 | 1 | 1 | 1 | 0 | 0 | 0 | 0 |
| 480 mg fasted | 17 | 16 | 11 | 9 | 9 | 9 | 7 | 6 | 5 | 5 | 4 | 4 | 4 | 4 | 4 | 4 | 4 | 3 | 0 | 0 |

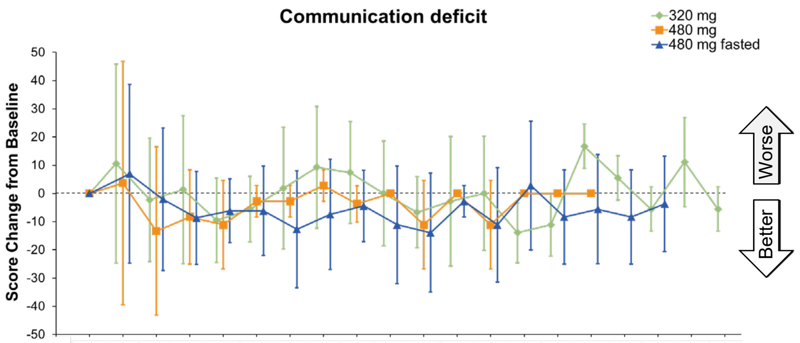


| Cycle | BL | C1 | C3 | C5 | C7 | C9 | C11 | C13 | C15 | C17 | C19 | C21 | C23 | C25 | C29 | C33 | C37 | C41 | C45 | C49 |
|---|---|---|---|---|---|---|---|---|---|---|---|---|---|---|---|---|---|---|---|---|
| 320 mg | 20 | 20 | 15 | 8 | 7 | 6 | 6 | 6 | 6 | 6 | 5 | 4 | 4 | 4 | 3 | 2 | 2 | 2 | 2 | 2 |
| 480 mg | 7 | 6 | 5 | 4 | 4 | 4 | 4 | 4 | 3 | 2 | 2 | 2 | 2 | 1 | 1 | 1 | 0 | 0 | 0 | 0 |
| 480 mg fasted | 17 | 16 | 11 | 9 | 9 | 9 | 7 | 6 | 5 | 5 | 4 | 4 | 4 | 4 | 4 | 4 | 4 | 3 | 0 | 0 |

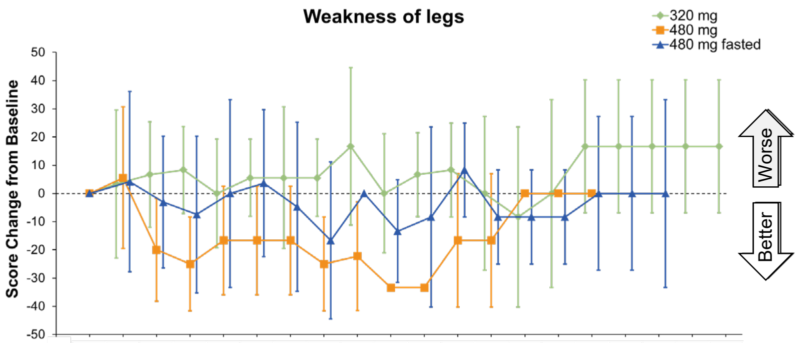


| Cycle | BL | C1 | C3 | C5 | C7 | C9 | C11 | C13 | C15 | C17 | C19 | C21 | C23 | C25 | C29 | C33 | C37 | C41 | C45 | C49 |
|---|---|---|---|---|---|---|---|---|---|---|---|---|---|---|---|---|---|---|---|---|
| 320 mg | 20 | 20 | 15 | 8 | 7 | 6 | 6 | 6 | 6 | 6 | 5 | 4 | 4 | 4 | 3 | 2 | 2 | 2 | 2 | 2 |
| 480 mg | 7 | 6 | 5 | 4 | 4 | 4 | 4 | 4 | 3 | 2 | 2 | 2 | 2 | 1 | 1 | 1 | 0 | 0 | 0 | 0 |
| 480 mg fasted | 17 | 16 | 11 | 9 | 9 | 9 | 7 | 6 | 5 | 5 | 4 | 4 | 4 | 4 | 4 | 4 | 4 | 3 | 0 | 0 |

# Supplementary Figure S7. See figure legend for the later page

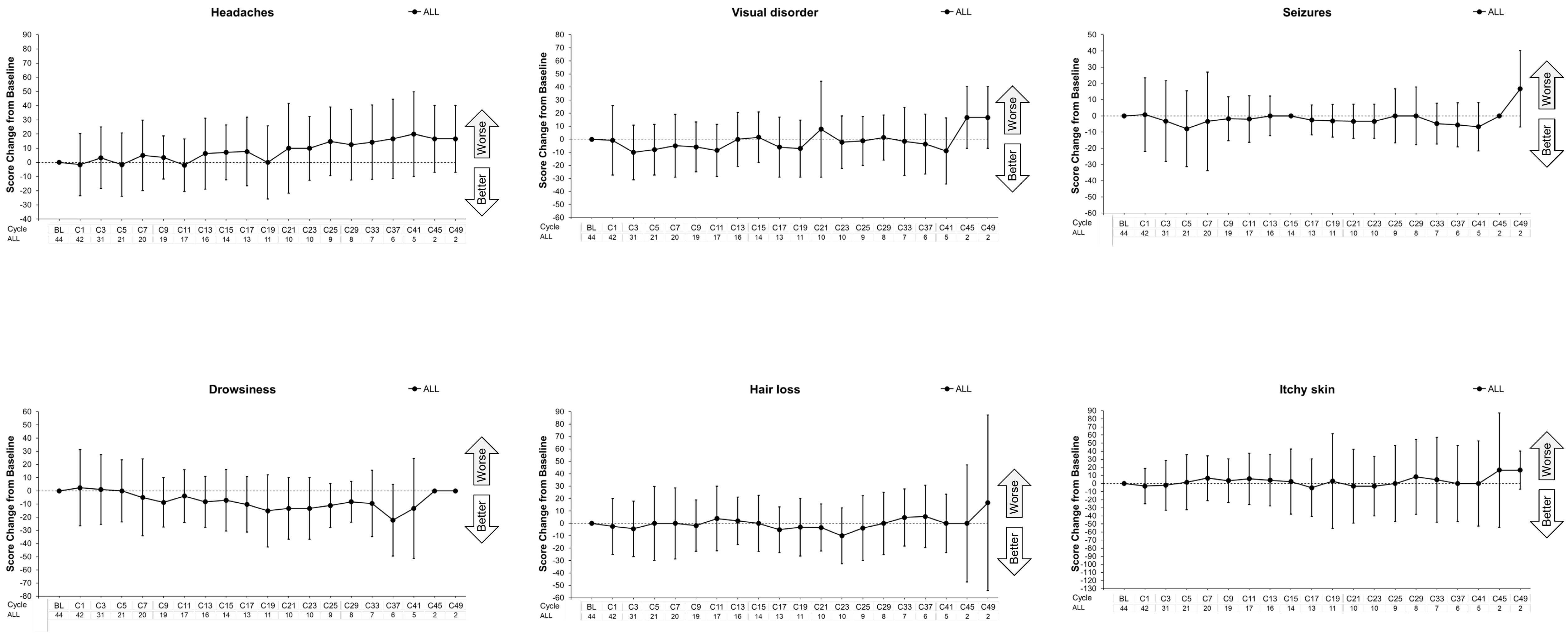

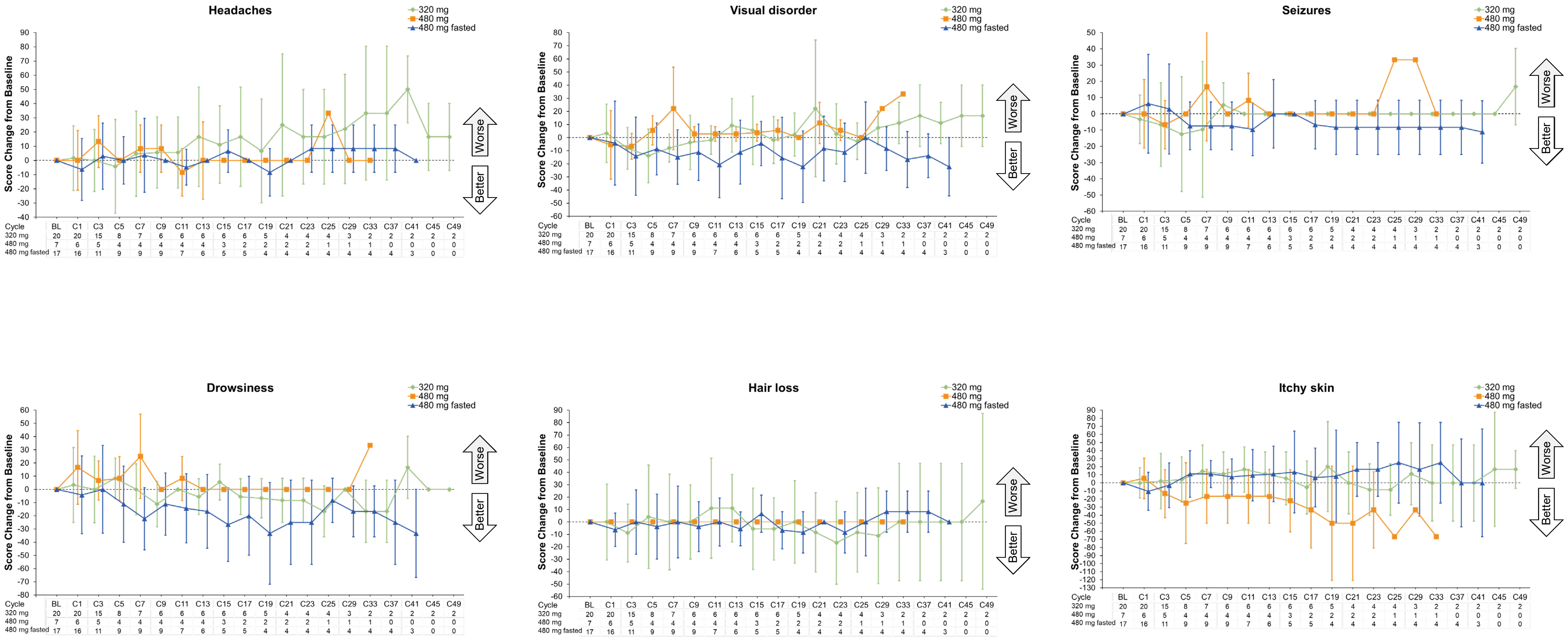


**Supplementary Figure S7. QLQ-BN20 in all patients and each dose group**

The average changes from baseline are shown. Error bars indicate the standard deviation. The horizontal dotted black lines show the baselines. **P* <0.05 in the Dunnett's test in comparison with the baselines. BL, baseline; QLQ-BN20, the core QoL questionnaires-BN20.

# Supplementary Figure S8. See figure legend for the later page

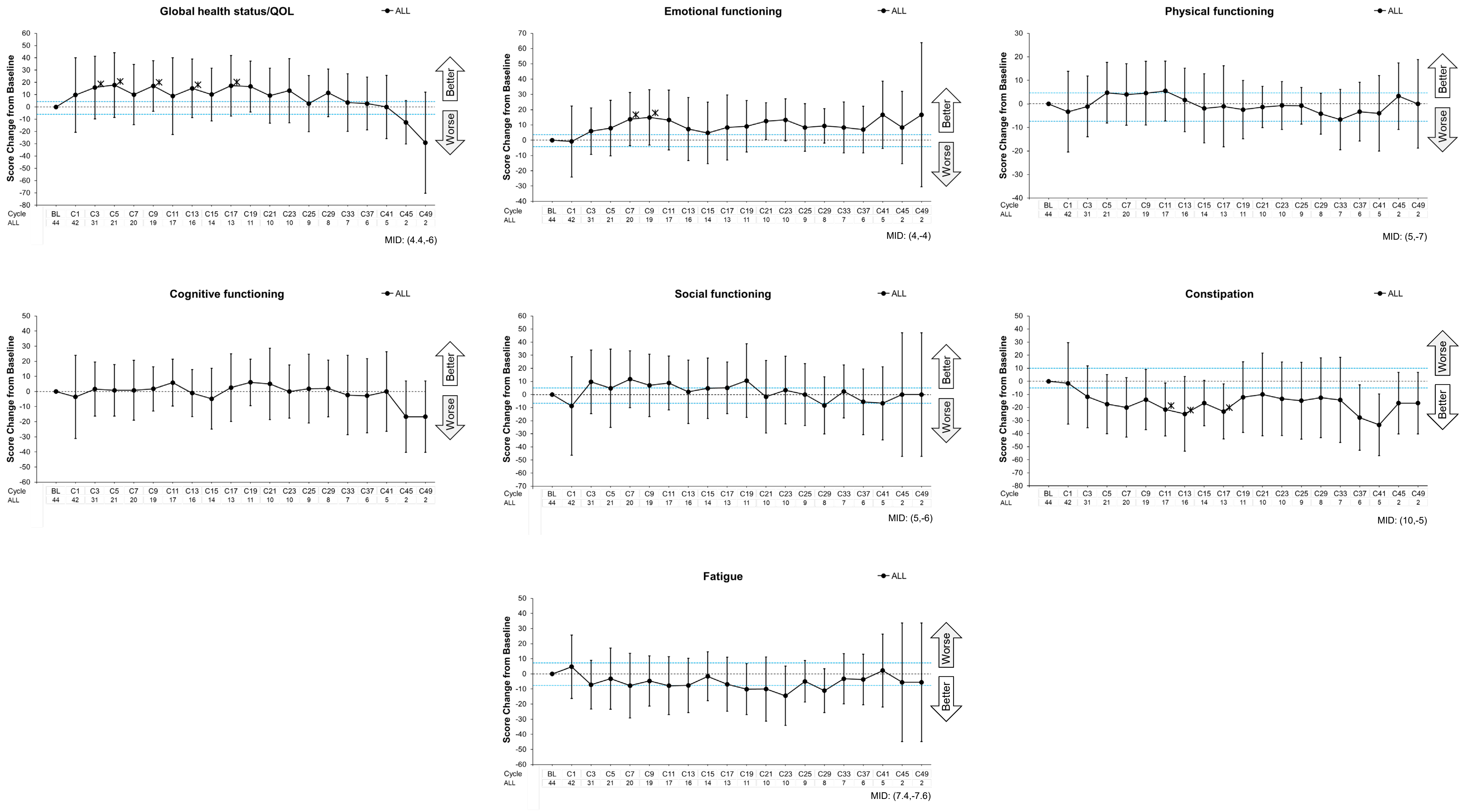

# Supplementary Figure S8. See figure legend for the later page

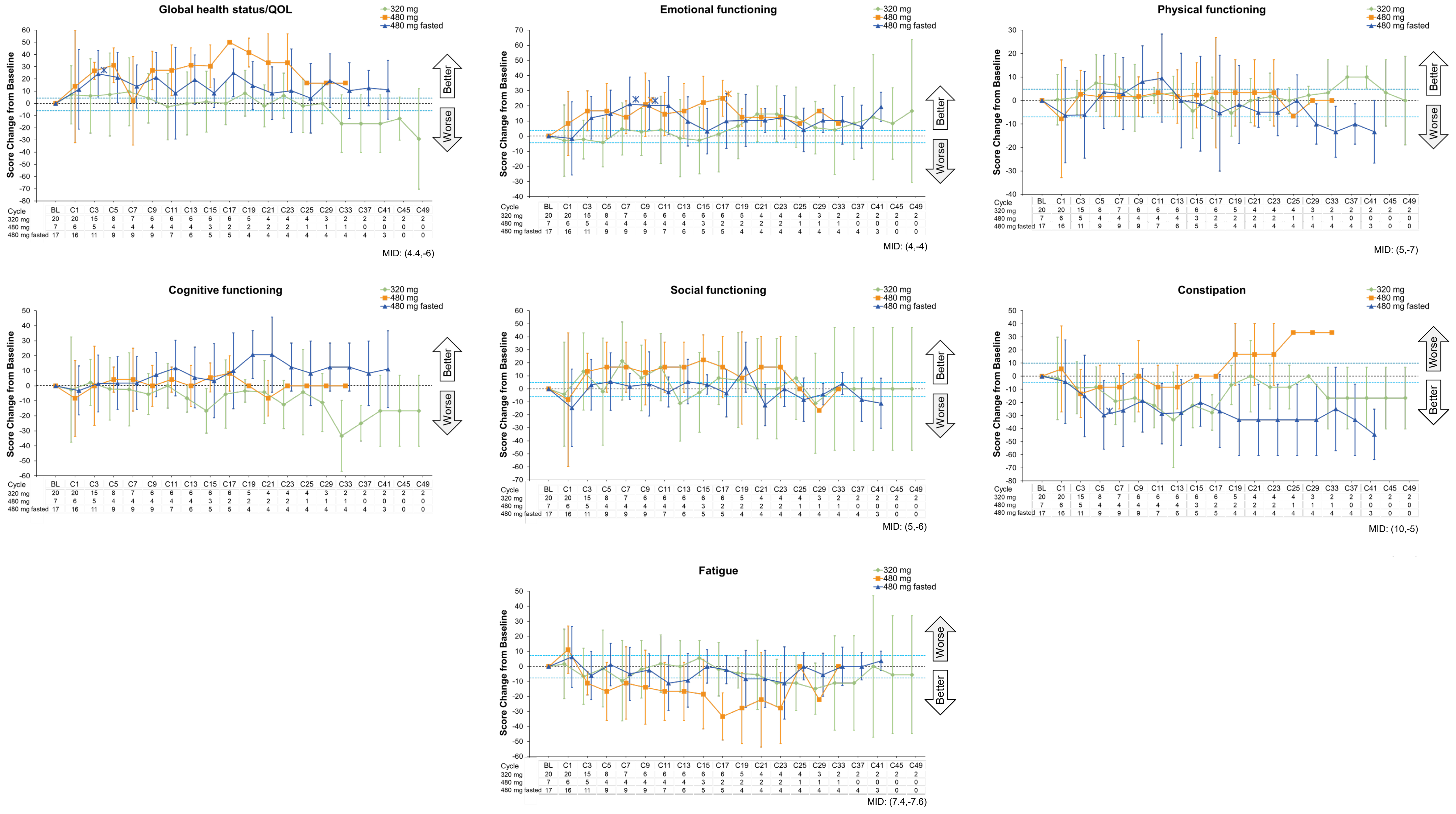

## Supplementary Figure S8. See figure legend for the later page

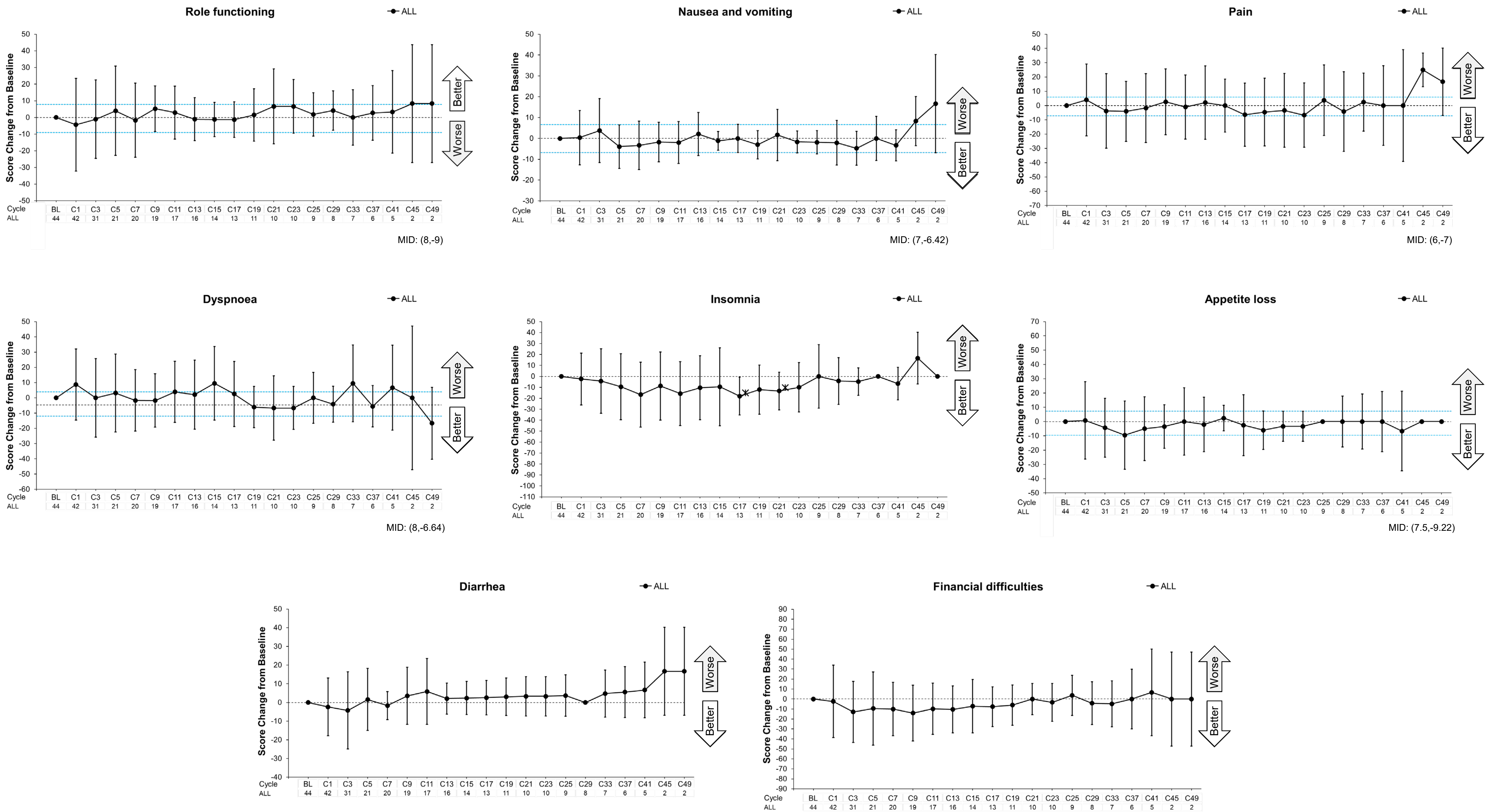

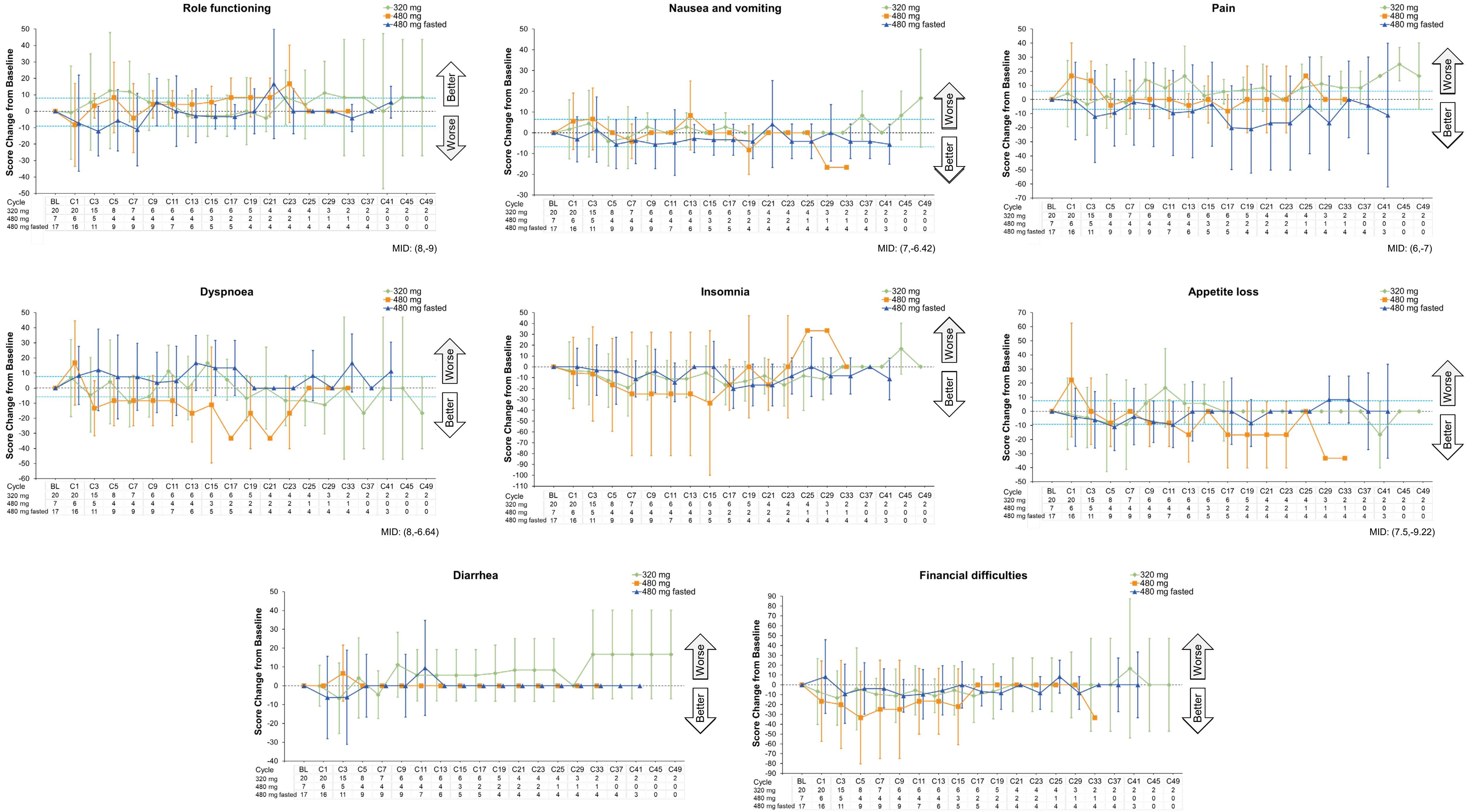


**Supplementary Figure S8. QLQ-C30 in all patients and each dose group**

The average changes from baseline are shown. Error bars indicate the standard deviation. The horizontal dotted black lines and dotted light blue lines show the baselines and minimally important differences, respectively. *$P$ <0.05 in the Dunnett's test in comparison with the baselines. BL, baseline; QLQ-C30, core QoL questionnaires-C30; MID, minimally important difference.

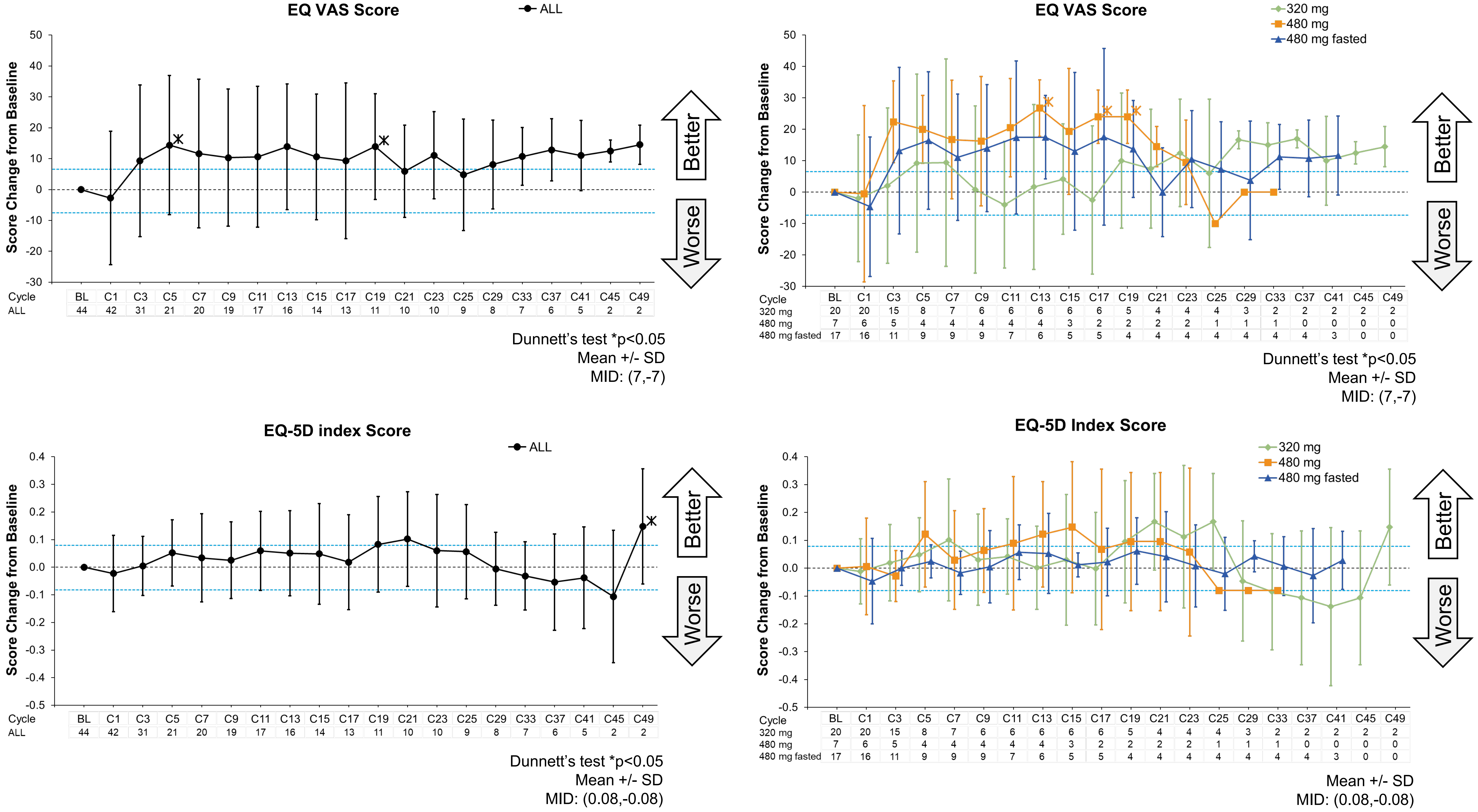


**Supplementary Figure S9. EQ-5D-3L scores in all patients and each dose group**

The average changes from baseline are shown. Error bars indicate the standard deviation. The horizontal dotted black lines and dotted light blue lines show the baselines and minimally important differences, respectively. *$P$ <0.05 in the Dunnett's test in comparison with the baselines. BL, baseline; EQ-5D-3L, the EuroQoL 5 dimensions 3-level; MID, minimally important difference; VAS, visual analog scales.
